# Roux-en-Y gastric bypass and sleeve gastrectomy induce substantial and persistent changes in microbial communities and metabolic pathways

**DOI:** 10.1080/19490976.2022.2050636

**Published:** 2022-03-22

**Authors:** Jerry T. Dang, Valentin Mocanu, Heekuk Park, Michael Laffin, Naomi Hotte, Shahzeer Karmali, Daniel W. Birch, Karen L. Madsen

**Affiliations:** a Department of Surgery, University of Alberta, Edmonton, Alberta, Canada; b Department of Medicine, Columbia University, New York, New York, USA; c Department of Medicine, University of Alberta, Edmonton, Alberta, Canada

**Keywords:** Metabolic surgery, bariatric surgery, intestinal physiology, microbiome, metabolomics, Roux-en-Y gastric bypass, sleeve gastrectomy

## Abstract

Bariatric surgery induces significant microbial and metabolomic changes, however, links between microbial and metabolic pathways have not been fully elucidated. The objective of this study was to conduct a comprehensive investigation of the microbial, metabolomic, and inflammatory changes that occur following Roux-en-Y gastric bypass (RYGB) and sleeve gastrectomy (SG). A prospective clinical trial was conducted with participants undergoing RYGB, SG, and non-operative controls (CTRL). Clinical parameters, blood samples, and fecal samples were collected pre-intervention and at 3 and 9 months. A multi-omics approach was used to perform integrated microbial-metabolomic analysis to identify functional pathways in which weight loss and metabolic changes occur after surgery. RYGB led to profound microbial changes over time that included reductions in alpha-diversity, increased Proteobacteria and Verrucomicrobiota, decreased Firmicutes, and numerous changes at the genera level. These changes were associated with a reduction in inflammation and significant weight loss. A reduction in *Romboutsia* genera correlated strongly with weight loss and integrated microbial-metabolomic analysis revealed the importance of *Romboutsia*. Its obliteration correlated with improved weight loss and insulin resistance, possibly through decreases in glycerophospholipids. In contrast, SG was associated with no changes in alpha-diversity, and only a small number of changes in microbial genera. A cluster of Firmicutes genera including *Butyriciccocus, Eubacterium ventriosum*, and *Monoglobus* was decreased, which correlated with decreased weight, insulin resistance, and systemic inflammation. This work represents comprehensive analyses of microbial-metabolomic changes that occur following bariatric surgery and identifies several pathways that are associated with beneficial metabolic effects of surgery.

## Introduction

The two most commonly performed bariatric surgical procedures are the Roux-en-Y gastric bypass (RYGB) and sleeve gastrectomy (SG)^1^. Patients with RYGB typically experience greater weight loss and have better glycemic control than those patients undergoing SG.^2^ The weight loss that occurs following these procedures is thought to occur due to multiple mechanisms, including reduced caloric intake, decreased nutrient absorption, increased satiety, increased release of satiety-promoting gut hormones (glucagon-like peptide 1, peptide YY) and shifts in bile acid metabolism.^3,4^

Recent evidence has linked changes in the gut microbiota to these beneficial effects of bariatric surgery. Studies have described changes in the composition and diversity of the gut microbiota that occur after RYGB and SG.^5–17^ However, differences in gut microbiota following these two different surgical procedures have not been well documented, and links between these microbial changes and metabolic pathways have not been well elucidated. The understanding of these relationships is essential to understanding the physiology of bariatric surgery, and in identifying therapeutic pathways through which bariatric surgery reverses metabolic dysfunction.

The aim of this study was to investigate and compare the microbial, metabolomic, and inflammatory changes that occur following RYGB and SG. Specifically, we used an integrated systems-based multi-omics approach to identify functional pathways associated with weight loss after bariatric surgery, to improve understanding of the physiological changes that occur with bariatric surgery. Understanding the role that alterations in microbial function may have in inducing weight loss following bariatric surgery has the potential to identify approaches involving microbial manipulation that could either potentiate or replicate the effect of surgery. We hypothesize that the altered intestinal physiology following surgery will lead to changes in specific microbial populations, and those changes will in turn contribute to alterations in metabolomic and inflammatory pathways that induce weight loss and improved metabolic dysfunction.

## Results

### Patient characteristics

Eighty patients (28 control, 23 SG, 29 RYGB) were recruited, with ten (3 control, 5 SG, 2 RYGB) being lost to follow up at 3 months. An additional four patients from the control (CTRL) group were lost at 9 months because they either underwent earlier bariatric surgery or were lost to follow-up (Supplementary Figure 1). Recruitment was discontinued early due to an increased preference to treat patients preoperatively with liraglutide by the bariatric team, which significantly reduced the number of eligible participants. Patient demographics are summarized in Table 1. There was a significantly lower baseline body mass index (BMI) in the surgical cohorts (p = .002). Patients undergoing bariatric surgery had a lower baseline weight because patients must complete a comprehensive weight loss program prior to being offered bariatric surgery and those who were more compliant with the program were more likely to be offered surgery.

**Table 1.** Patient baseline demographics

Demographics mean (SD) or n (%)	Non-operative controls n = 25	Sleeve gastrectomy n = 18	Roux-en-Y gastric bypass n = 27	p-value
Number of patients (n)	25	18	27	-
Age at surgery (years)	47.7 (8.7)	47.9 (9.7)	47 (9.9)	0.940
Sex (female)	20 (80.0%)	17 (94.4%)	25 (92.6%)	0.356
Height (m)	1.67 (0.07)	1.69 (0.07)	1.67 (0.08)	0.725
Body mass index (kg/m^2^)	47.1 (7.3)	40.8 (5.7)	42.9 (4.2)	0.002
General anxiety disorder	7 (28.0)	10 (55.6)	11 (40.7)	0.194
Coronary artery disease	0 (0.0)	0 (0.0)	0 (0.0)	1.000
Depression	13 (52.0)	10 (55.6)	15 (55.6)	0.982
Type 2 diabetes	2 (8.0)	1 (5.6)	3 (11.1)	0.877
Dyslipidemia	8 (32.0)	8 (44.4)	6 (22.2)	0.301
Gastroesophageal reflux	8 (32.0)	8 (44.4)	13 (48.2)	0.505
Hypertension	8 (32.0)	6 (33.3)	10 (37.0)	0.949
Hypothyroidism	4 (16.0)	5 (27.8)	6 (22.2)	0.661
Fatty liver disease	4 (16.0)	5 (27.8)	5 (18.5)	0.649
Osteoarthritis	12 (48.0)	9 (50.0)	9 (33.3)	0.444
Polycystic ovarian syndrome	1 (4.0)	3 (16.7)	2 (7.4)	0.428
Asthma	4 (16.0)	2 (11.1)	5 (18.5)	0.916
Obstructive sleep apnea	11 (44.0)	10 (55.6)	15 (55.6)	0.718
Ex-smoker	8 (32.0)	1 (5.6)	6 (22.2)	0.120
EOSS 0 1 2 3	0 (0.0) 2 (8.0) 21 (84.0) 2 (8.0)	0 (0.0) 1 (5.6) 16 (88.9) 1 (5.6)	3 (11.1) 3 (11.1) 20 (74.1) 1 (3.7)	0.625

EOSS, Edmonton obesity staging system.

### Medications changes and postoperative complications

Patients in the CTRL cohort did not have any significant changes to medications during the study period. The SG cohort had four patients discontinue antihypertensives and two patients discontinue metformin postoperatively, while the RYGB cohort had eight patients discontinue antihypertensives, four patients discontinue metformin, and one patient discontinues gliclazide postoperatively.

There were no complications after SG in any patients. Three patients in the RYGB cohort had complications, including marginal ulcer, postoperative bleeding requiring transfusion, and early dumping syndrome.

### Body mass index

Body mass index (BMI) decreased significantly after SG and RYGB at 3- and 9-month time points, while CTRL subjects did not demonstrate any significant weight loss (Supplementary Figure 2). At 9 months, the mean BMI change after RYGB was −11.2 kg/m^2^ (p < .0001) and after SG was −8.2 kg/m^2^ (p < .0001).

### Clinical biochemistry

Clinical biochemical results are summarized in Supplementary Table 1. There were significant improvements in lipid profiles after RYGB at 3 and 9 months (Supplementary Figure 3). There were also significant improvements in glucose metabolism after both SG and RYGB at 3 and 9 months. This included lower fasting blood glucose, hemoglobin A1c (HbA1c), fasting serum insulin, and homeostatic model of insulin resistance (HOMA-IR) (Supplementary Figure 4).

### Inflammatory markers, lipopolysaccharide, and interleukins

There was a significant and progressive reduction in inflammatory markers after RYGB at 3 and 9 months. This included decreased C-reactive protein (CRP), white blood cells, and ferritin. SG did not have changes in CRP or ferritin but did have a significant decrease in white blood cells at 9 months. Lipopolysaccharide (LPS), as a measure of gut barrier integrity, did not show any significant changes at 3 or 9 months in any cohort (Supplementary Figure 5, Supplementary Table 1). There were no differences in cytokines (IL-1β, IL-6, IL-8, IL-10, or TNF-α) between timepoints in any of the groups (Supplementary Figure 6, Supplementary Table 2).

### Microbial alpha- and beta-diversity between timepoints

**Figure 1.**
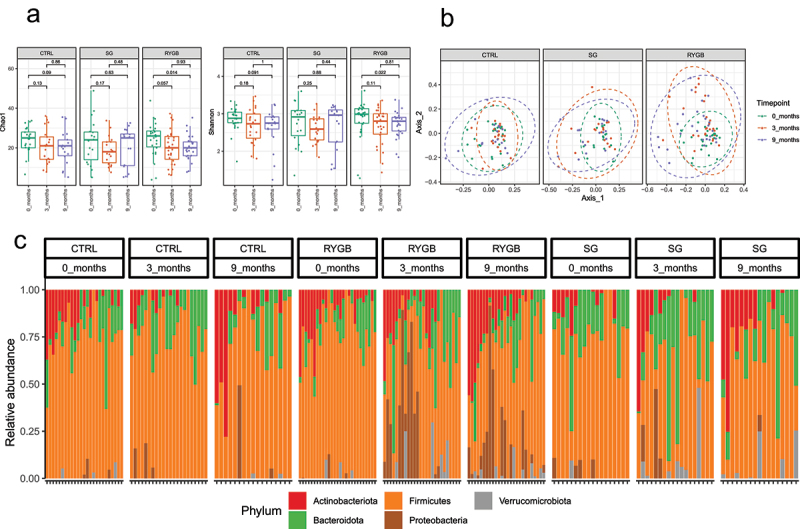
Differences in relative microbial abundance between non-operative control, sleeve gastrectomy and Roux-en-Y gastric bypass 0, 3, and 9 months. (a) Between timepoint differences in α diversity using the Chao1 and Shannon indices. (b) Between timepoint differences in β diversity using the Bray-Curtis dissimilarity index. (c) Taxa bar plots demonstrating phylum level differences in relative microbial abundance between groups.

RYGB had a statistically significant decrease in alpha-diversity at 9 months compared to baseline as demonstrated by lower Shannon and Chao1 indices (Figure 1a), while CTRL and SG did not demonstrate any changes in alpha-diversity. Similarly, significant changes in beta-diversity (Bray-Curtis) were only present for the RYGB cohort when comparing baseline and 3-month (p = .002) and 0 and 9-month (p = .008) time points. CTRL and SG did not demonstrate any differences (p > .05) (Figure 1b).

### Differences in microbial abundance between timepoints

At the phylum level, patients undergoing RYGB demonstrated the most dramatic shifts in microbial composition, with an increase in Proteobacteria and Verrucomicrobiota and a loss of Firmicutes at 3 months. At 9 months, these changes were sustained and included an increase in the abundance of Desulfobacterota. At the genus level, at 3 months there were increases in *Actinomyces, Streptococcus, Veillonella, Ruminococcaceae NK4A214, UCG-005, Akkermansia, Escherichia-Shigella* and decreases in *Dorea, Eubacterium ventriosum, Fusicatenibacter, Coprococcus, Erysipelotrichaceae, UCG-003, Anaerostipes, Eubacterium hallii, Christensenellaceae R-7* group, *Ruminococcus torques, UCG-002* and *Blautia*. At 9 months, there were increases in *Veillonella, NK4A214, Streptococcus, Anaerotruncus, Escherichia-Shigella, UCG-005, Akkermansia, Klebsiella* and decreases in *Anaerostipes, Oscillibacter, Dorea, Eubacterium ventriosum, Family XIII UCG-001, Romboutsia, Faecalibacterium, Lachnospira*, and *Dialister* (Supplementary Figures 7 to 18).

A different microbial profile was seen in patients undergoing SG. This cohort did not have significant microbial changes at 3 months but had an increase in the abundance of Actinobacteriota with a loss of Proteobacteria and Bacteroidota at 9 months. There were also differences in the SG cohort at the genus level at 9 months including increases in *Streptococcus* and decreases in *Monoglobus, Agathobacter, Butyricicoccus, Eubacterium hallii*, and *Lachnospiraceae UCG-010*.

As expected, the control cohort had no significant differences at either the phylum or genus levels at 3 and 9 months compared to baseline (Figure 1c).

### Correlations between microbial taxa and clinical parameters

The RYGB cohort demonstrated mixed correlations between Firmicutes species and metabolic parameters. Within the Firmicutes phyla, *Butyricicoccus, Erysiplotrichaceae, UCG-003, Monoglobus, Dialister, Lachnospira, Oscillibacter, Romboutsia, Anaerostipes*, and *Eubacterium ventriosum* had positive correlations with metabolic parameters, while *UCG-002, Veillonella, Christensenellaceae R-7* group, *NK4A214, Streptococcus, Alistipes*, and *Collinsella* had negative correlations. Notably, genera within *Proteobacteria* were negatively associated with multiple metabolic parameters (HOMA-IR, fasting serum insulin, low-density lipoprotein, triglycerides, and total cholesterol) and CRP and *Akkermansia* had negative correlations with weight, insulin resistance, and CRP (Supplementary Figure 19b).

The SG cohort demonstrated positive correlations between multiple Firmicutes microbes including *Butyricicoccus, Lachnospiraceae UCG-010, Eubacterium ventriosum, CAG-56* and metabolic parameters including higher weight, fasting blood glucose, HbA1c, and fasting serum insulin but lower high-density lipoproteins (HDL). *Streptococcus* was the only Firmicutes bacteria with negative correlations to metabolic parameters and positive correlations to HDL (Supplementary Figure 19a).

### Integrated microbiome–metabolome analysis

In the RYGB and SG cohorts, integrated microbiome–metabolome analysis demonstrated significant discrimination between baseline and 9 months in both cohorts with RYGB showing greater levels of separation. There was limited discrimination seen in CTRL at 0, 3, and 9 months (Supplementary Figure 20).

**Figure 2.**
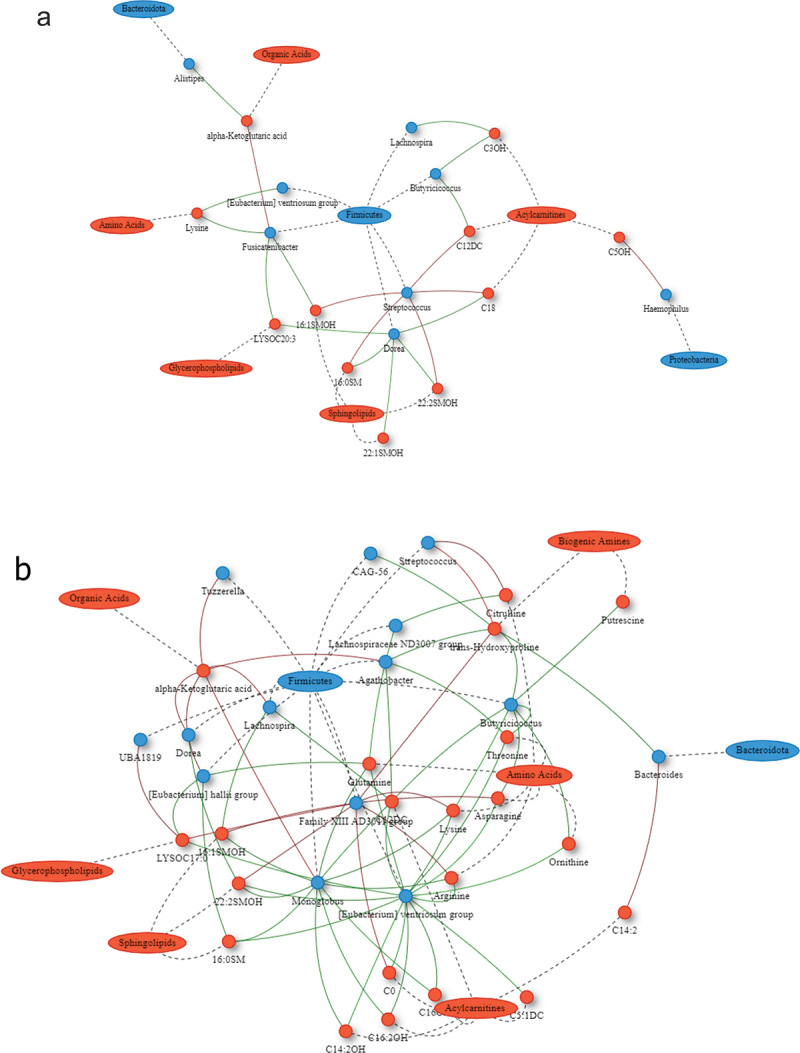
Network plot of Spearman correlations between differential microbes and metabolites at 3 and 9 months compared to baseline for sleeve gastrectomy. Metabolites are represented as red circles and metabolite classes as red ovals. Microbial genera are represented as blue circles and phyla as blue ovals. Positive and negative correlations are indicated using red and green colors, respectively. SM, sphingomyelins; SMOH, hydroxysphingomyelin; PC, phosphatidylcholine; LYSOC, lysophosphatidylcholine; C, carnitines.

**Figure 3.**
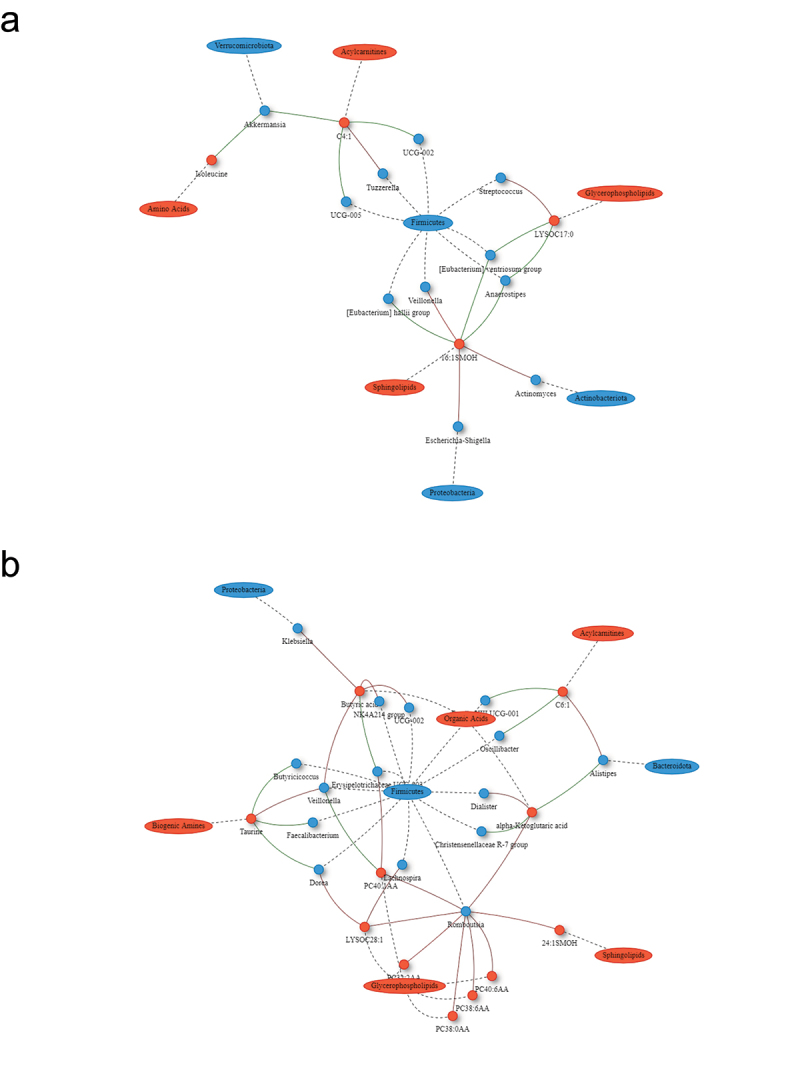
Network plot of Spearman correlations between differential microbes and metabolites at 3 and 9 months compared to baseline for Roux-en-Y gastric bypass. Metabolites are represented as red circles and metabolite classes as red ovals. Microbial genera are represented as blue circles and phyla as blue ovals. Positive and negative correlations are indicated using red and green colors, respectively. SM, sphingomyelins; SMOH, hydroxysphingomyelin; PC, phosphatidylcholine; LYSOC, lysophosphatidylcholine; C, carnitines.

The SG and RYGB cohorts demonstrated an increasing number and complexity of microbial-metabolomic interactions from 3 to 9 months after bariatric surgery. The 9-month SG cohort demonstrated the most interactions, with a cluster of Firmicutes bacteria (*Butyricicoccus, Eubacterium ventriosum*, and *Monoglobus*) having negative correlations with metabolites of various classes including amino acids, sphingolipids, and acylcarnitines (Figure 2). There were also negative correlations between sphingolipids and Firmicutes microbes including *Monoglobus, Eubacterium ventriosum, Eubacterium hallii, Dorea*, and *Lachnospira*. RYGB at 9 months was also dominated by interactions through Firmicutes bacteria with *Romboutsia* having positive correlations with multiple glycerophospholipid metabolites (Figure 3). The CTRL group demonstrated minimal correlations.

**Figure 4.**
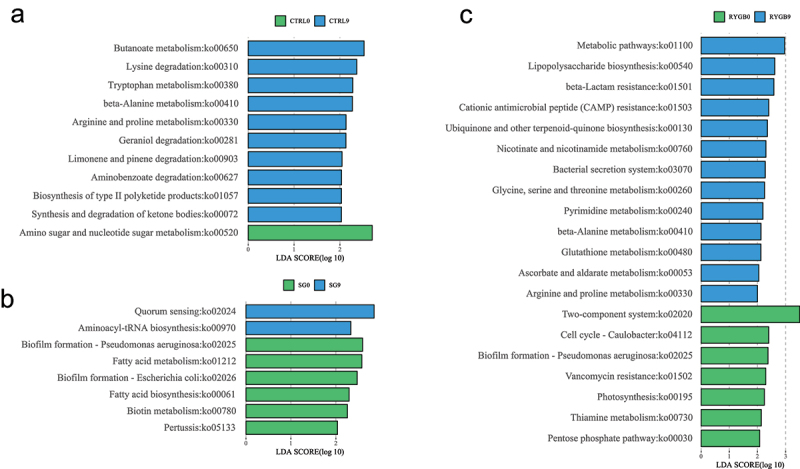
Microbial functional prediction of KEGG-based KO functions using linear discriminant analysis comparing 9 months to baseline for (a) non-operative control, (b) sleeve gastrectomy, and (c) Roux-en-Y gastric bypass. Green represents enriched pathways at baseline and blue represents enriched pathways at 9 months. P-value < 0.05 is considered statistically significant.

### Microbial functional prediction and metabolic pathway enrichment analysis

**Figure 5.**
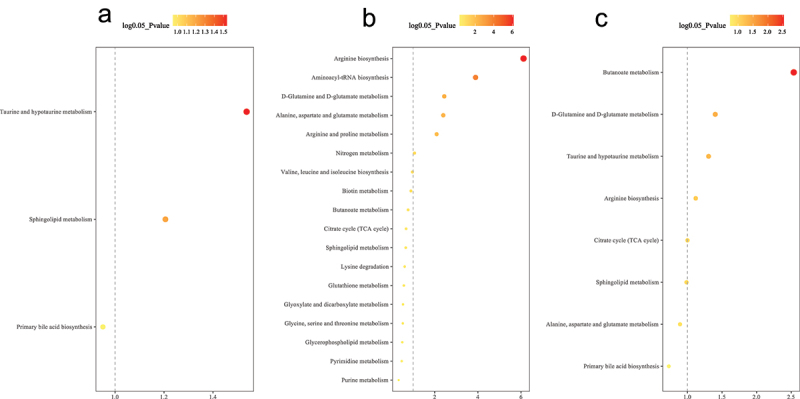
Differential metabolic pathway enrichment analysis comparing 9 months to baseline for (a) non-operative control, (b) sleeve gastrectomy, and (c) Roux-en-Y gastric bypass. P-value < 0.05 (or log 0.05 p-value > 1.0) is considered statistically significant.

Differentially enriched and depleted KEGG-orthology (KO) functional pathways were identified between baseline and 9 months. RYGB had the most significant changes with 20 microbial and four metabolomic functions identified (Figure 4, Figure 5). The SG cohort had differentially significant changes in eight microbial and six metabolic KO functional pathways, while the CTRL cohort, 11 microbial and two metabolomic pathways were identified.

**Figure 6.**
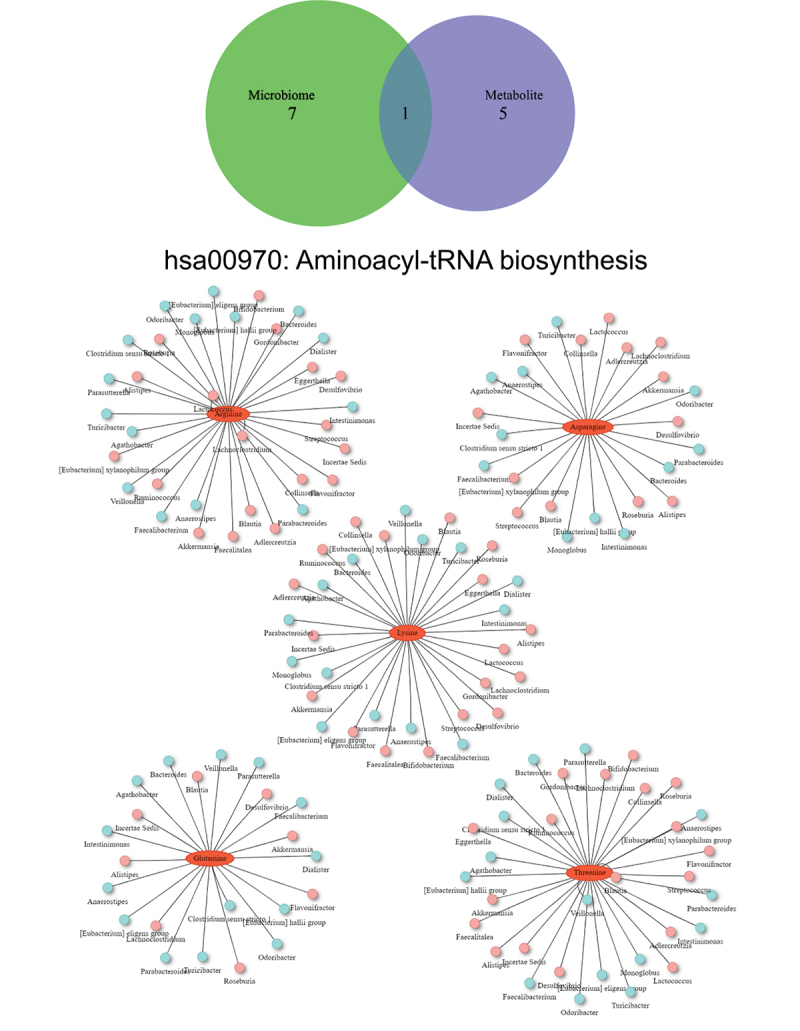
Venn diagram of significant differential pathways in functional network analysis implemented with the KEGG metabolic pathway database. Interaction network plots of enriched amino acids in the aminoacyl-transfer-RNA biosynthesis pathway with microbes that participate in its metabolism. Red colors indicate significantly up-regulated metabolites or microbes, while blue colors indicate significantly down-regulated metabolites or microbes.

Among all group comparisons, only the 9-month SG cohort demonstrated a common enriched functional pathway on both microbial and metabolic functional analysis. The aminoacyl-transfer-RNA (aa-tRNA) biosynthesis pathway was significantly upregulated in both microbial and metabolomic functional pathways. This pathway was specifically enriched by five amino acids (arginine, asparagine, lysine, glutamine, threonine) and modified by potentially 79 unique bacterial genera (Figure 6).

### Between group comparisons between SG and RYGB

On univariate microbial analysis, at 3 months, there were no significant microbial differences between RYGB or SG. However, at 9 months, RYGB had a higher abundance of Proteobacteria compared to SG but no differences at the genus level (Supplementary Figure 21 to 24). Sparse partial least squares discriminant analysis (sPLS-DA) score plots demonstrated significant discrimination between RYGB and SG at 3 months; however, this difference was not significant at 9 months (Supplementary Figure 25).

**Figure 7.**
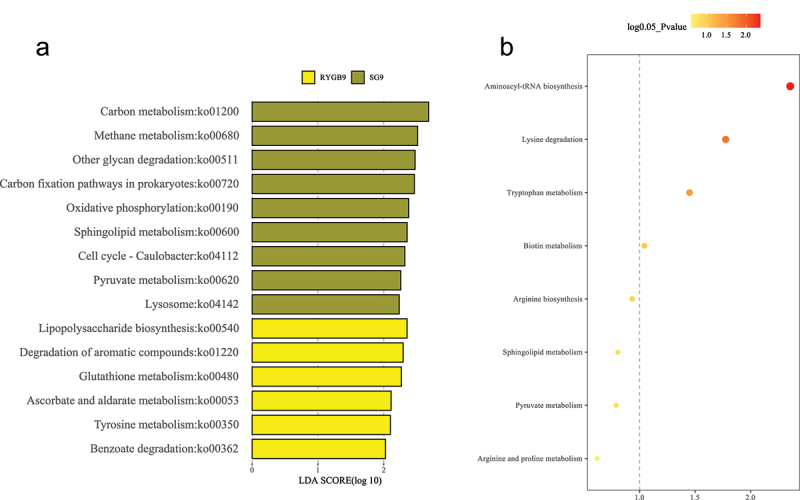
Functional analysis between sleeve gastrectomy and Roux-en-Y gastric bypass at 9 months for (a) microbial pathways and (b) metabolomic pathways. Bright yellow represents enriched pathways for Roux-en-Y gastric bypass and dark yellow represents enriched pathways for sleeve gastrectomy. P-value < 0.05 (or log 0.05 p-value > 1.0) is considered statistically significant.

Microbial functional pathway analysis revealed nine enriched pathways in SG and six enriched pathways in RYGB. In metabolomic pathway enrichment analysis, the aminoacyl-tRNA biosynthesis pathway was the most significant metabolomic pathway enriched in SG compared to RYGB. Sphingolipid metabolism was significantly enriched in microbial functional analysis (p = .03) and had near significance in metabolomic pathway enrichment analysis (p = .09) in SG compared to RYGB (Figure 7).

## Discussion

This study represents the largest trial to date with a comprehensive and integrated analysis of microbial, metabolomic, inflammatory, and clinical changes between patients receiving aggressive medical intervention for obesity and those receiving the two most common bariatric surgical interventions. RYGB and SG cohorts both achieved significant postoperative weight loss. Additionally, RYGB led to improvements in metabolic and inflammatory measures, and these were associated with numerous significant microbial changes. SG produced modest microbial shifts but had pronounced metabolomic changes. In contrast, the CTRL cohort had minimal changes in clinical, inflammatory, microbial, or metabolomic parameters. This prospective clinical trial and bioinformatics analysis revealed unique pathways in which weight loss and metabolic improvement occurred after bariatric surgery.

RYGB led to a consistent decrease in markers of systemic inflammation, including temporal decreases in CRP, WBC, and ferritin. This was despite the microbial composition shifting toward purportedly pro-inflammatory and pathologic bacterial phyla Proteobacteria, and genera including Escherichia-Shigella and Klebsiella.^18^ The mechanism for increased Proteobacteria following RYGB is thought to be secondary to an alkalized environment of the proximal enteral tract due to exclusion of the acid-producing stomach after RYGB. Proteobacteria are less acid adaptive than other phyla and increasingly alkaline environments encourage their proliferation.^19^ Increased oxygen within the intestinal lumen after RYGB may also contribute to this proliferation since Proteobacteria includes many species that produce enzymes such as catalase and superoxide dismutase that can neutralize reactive oxygen species.^20,21^

Glycerophospholipids are a group of molecules thought to be involved in obesity and insulin resistance. They have been found to be increased in the myotubes of obese patients, and membrane glycerophospholipid dynamics are linked to the development of diet-induced insulin resistance.^22^ The *Romboutsia* genus, which was profoundly depressed after RYGB, was strongly linked with a decrease in six different glycerophospholipids. Further, depletion of *Romboutsia* correlated with weight loss and decreased insulin resistance, a finding which has been previously described.^23,24^ Other works have linked *Romboutsia* to glycerophospholipids, which have been implicated in obesity-induced fatty liver disease.^25^ Together, these findings suggest a role for *Romboutsia* in the modulatory effects of RYGB on glycerophospholipids.

SG produced less extensive microbial and metabolic alterations than RYGB. Although impressive weight loss was noted, microbial and metabolic changes in SG centered around one Firmicutes cluster consisting of *Butyriciccocus, Eubacterium ventriosum*, and *Monoglobus* (*BEM*), which had negative correlations with a wide array of metabolites. This cluster decreased in abundance after SG, a change that was associated with an increase in various metabolites including amino acids, acylcarnitines, and sphingolipids. Previous works have identified the importance of these bacterial clusters in obesity-related disease. *Eubacterium ventriosum* has been associated with obesity,^26,27^ while butyrate-producing organisms such as *Butyricicoccus* appear to cause shifts in fermentation patterns which affect energy homeostasis.^27^ Our work identified this cluster as being associated with higher weight, higher insulin resistance, higher fasting blood glucose, and greater systemic inflammation.

These effects may be mediated through the aa-tRNA biosynthesis pathway, which was enriched in both microbial and metabolic functional analysis. After SG, this pathway is driven by increases in five amino acids that were associated with a reduction in the abundance of *BEM* bacteria. tRNAs are formed by direct aminoacylation of tRNAs, which are catalyzed by aminoacyl-tRNA synthetases (aaRS).^28^ Alterations in tRNA biology have been associated with metabolic disorders.^29^ Specifically, mutations in aaRSs and variants of the tRNA-modifying enzyme CDKAL1 have been associated with an increased risk of obesity and type 2 diabetes.^30–32^ Mutations in mitochondrial tRNA genes have also been associated with maternally inherited diabetes,^33^ while mutations in tRNA methyltransferase TRMT10A directly cause young-onset diabetes.^34^ aaRSs are also involved in intracellular amino acid signaling and recent studies support the notion that depletion or enrichment of amino acids modulate the activity of aa-tRNA biosynthesis.^35^ Given the findings of our study, it is plausible that the loss of the *BEM* Firmicutes cluster encourages the production of amino acids, which concomitantly enrich the aa-tRNA biosynthesis pathway. Furthermore, this enrichment potentiates improved glucose, lower weight, and decreased systemic inflammation.

This work offers the largest direct systems-based comparison of SG to RYGB to date. The proportion of fecal Proteobacteria differed significantly between RYGB and SG, and as previously discussed, has been associated with changes in barrier function, weight loss, and systemic inflammation.^14,36^ SG also had distinctive enrichment of the sphingolipid metabolism pathway compared to RYGB within the microbiome and metabolome, including a loss of a cluster of Firmicutes encompassing the genera of *Monoglobus, Eubacterium ventriosum, Eubacterium hallii, Dorea*, and *Lachnospira*, which were correlated with increased serum sphingomyelins and hydroxysphingomyelins. A depletion in sphingomyelins, which are an important component of the cell membrane, is associated with diabetes^37^ and the impairment of pancreatic β-cell function.^38^ These divergences in microbial and metabolic pathways suggest the mechanism of weight loss, decreased inflammation, and improved metabolic parameters differ between SG and RYGB.

Limitations in the trial design include differences in baseline demographics, which are inherent in non-randomized prospective trials which may influence results. It is also possible that changes in medications, diet, and exercise may confound our findings. For example, some surgical patients had metformin discontinued postoperatively and there is evidence that metformin use independently alters the gut microbiota.^39^ Single doses of preoperative antibiotics may also induce changes to microbial compositions, although this effect is expected to be minimal and transient.^40^ Notably, patients were managed by the same clinical team with standardized preoperative and postoperative care, which should reduce confounding effects. We also considered the possibility that performing multiple analyses can impose a risk of false discovery; however, our control group underwent the same analysis and had minimal significant findings. Recruitment was also prematurely discontinued, and this potentially increases our risk for a type II error. However, despite these limitations, our study provides the most comprehensive analysis of the complex microbial–metabolomic relationships in bariatric surgery to date and identified pathways that may be the future target of therapeutic strategies for the treatment of obesity and metabolic disease.

In this prospective clinical trial, we performed a comprehensive analysis on the microbial, metabolomic, and inflammatory changes that occur with the RYGB and SG. Both procedures were associated with significant microbial and metabolic changes. Future works can build on these analyses by targeting modification of these microbial populations, and their resulting metabolites, which may lead to novel therapeutic strategies for the treatment of obesity and metabolic disease both in the context of medically managed and surgical patients.

## Patients and methods

### Study design

This study was designed as a three-arm parallel prospective interventional clinical trial with patients in RYGB, SG, and non-operative control (CTRL) cohorts. Patient demographics including height, weight, body mass index (BMI), and comorbidities were documented. Fecal, and blood samples were collected in clinic 4 weeks prior to surgery. In the post-operative period, blood and fecal collection took place at 3 and 9 months. All pre-operative measurements and samples were collected prior to subjects initiating a two to 3-week pre-operative liquid diet.

CTRL patients were treated with standardized diet, exercise, and behavioral interventions for weight loss. These patients were assessed and managed by a multidisciplinary team including obesity medicine physicians, registered nurses, registered dietitians, psychologists, and physical therapists. Patients underwent regular weight management educational workshops. Patients who received meal replacement or pharmacologic interventions for weight loss were excluded from the study. For this cohort, subjects had initial sampling prior to initiating weight loss interventions. Further sampling occurred at 3 months and 9 months following initiation of the intervention.

### Study objective

The primary objective of this study was to determine changes in microbial species and metabolites after SG and RYGB in relation to important metabolic parameters: weight, fasting blood glucose, HbA1c, fasting serum insulin, insulin resistance as estimated by HOMA-IR, lipids, and CRP.

### Study population

This study was approved by the Health Research Ethics Board at the University of Alberta (PRO00071705) and registered with ClinicalTrials.gov (NCT03181347) on June 8, 2017. Patients were recruited from the Edmonton Adult Specialty Bariatric Clinic from September 2017 to May 2019. The intent was to recruit 30 participants with a BMI greater than 35 kg/m^2^ into each arm including 30 CTRL, 30 SG, and 30 RYGB. Exclusion criteria included antibiotic, liraglutide, semaglutide, or methotrexate usage within 2 months preceding enrollment as these have significant effects on the gut microbiota. Additionally, patients with meal replacement use within 1 month, previous bowel surgery, inflammatory bowel disease, or previous bariatric surgery were excluded.

### Sample size calculation

Sample size calculations were performed a priori and designed to ensure we would adequately capture microbial changes induced by surgery. In prior literature, an important short-chain fatty acid-producing bacterial species’ (*F. prausnitzii*) relative abundance was lower in a post-RYGB group compared to non-operative controls (0.031 v. 0.053 σ 0.024).^12^ With an alpha of 0.05 and a beta of 0.90, this would require 26 subjects per arm. Including a dropout rate of 10%, this increases to 30 subjects per arm.

### Bariatric surgery procedures

Primary laparoscopic bariatric surgery was performed by three fellowship-trained bariatric surgeons. Patients received a single dose of weight-adjusted antibiotics 30 minutes prior to surgery. Patients undergoing SG received cefazolin while RYGB of cefazolin and metronidazole. SG was performed using a 50 French bougie with stapling tight to the bougie. RYGB was performed with a 110 cm Roux limb, 40 cm biliopancreatic limb, stapled jejunojejunostomy, and circular-stapled gastrojejunostomy. The Roux limb was placed antecolic, and the jejunostomy–jejunostomy mesenteric defect was routinely closed. Petersen’s defect was closed routinely by two surgeons.

### Clinical biochemistry

Blood samples were collected after a 12-hour fast. Plasma and serum were tested using Alberta Health Services Laboratory Services, a public-health laboratory system. This included fasting blood glucose, HbA1c, fasting serum insulin, and lipid panel. HOMA-IR was calculated from fasting blood glucose and insulin using the University of Oxford HOMA2 Calculator.^41^

### C-reactive protein, lipopolysaccharide, and inflammatory cytokines

Serum was assessed for CRP as a measurement of systemic inflammation, and LPS, as a measurement of bacterial translocation. Cytokines analyzed included IL-1β, IL-6, IL-8, IL-10, and TNF-α using enzyme-linked immunosorbent assays (R&D Systems, DuoSet for cytokines, Abbexa, abx514093 for LPS).

### Serum metabolomics

Metabolomics profiling was performed by liquid chromatography with tandem mass spectrometry targeting 143 metabolites by the Metabolomics Innovation Center using the Biocrates AbsoluteIDQ p180 kit.

### Fecal microbial analysis

Collection cups were provided to participants, and they were instructed to collect fecal specimen the night prior or morning of their appointment. Participants were instructed to store the specimen in the fridge in the interim and to transport them on ice to their appointment.

The microbial community composition of fecal samples was assessed using 16S rRNA gene analyses. DNA was extracted from fecal homogenates combining enzymatic and mechanical cell lysis with the DNA Stool Mini Kit (Qiagen, Valencia, CA, USA). Enteric microbiota composition was characterized by 16S rRNA tag sequencing using the MiSeq Illumina technology (pair-end), targeting the V3-V5 regions. This analysis was performed by Genome Quebec (Montreal, Canada).

Demultiplexed FASTQ 16S rRNA sequences were quality filtered, trimmed, dereplicated, and filtered for chimeric sequences using pair-ended DADA2 resulting in exact sequence variant (feature) tables.^42^ The table was imported into R 3.6.1 to analyze for α-diversity (Shannon/Chao1), β-diversity (wunifrac) and were performed using a function of the phyloseq v1.28.0 package.^43^ Ordination plots for β-diversity metrics were generated by non-parametric multidimensional scaling ordination in R.

### Statistical analysis

Baseline differences between groups were evaluated by univariate analyses using Fisher’s exact test for categorical data and one-way analysis of variance (ANOVA) for continuous data. Multiple comparisons were adjusted using the Benjamin–Hochberg method. Analyses were conducted using STATA 15 (StataCorp 2017; College Station, TX). Figures were designed using Prism 9.0.2 (GraphPad Software, San Diego, CA). Statistical significance was defined using two-tailed tests with a p-value <0.05. Error bars on figures represent standard error of the means and asterisks represent statistical significance with * as p < .05, ** as p < .01, *** as p < .001, **** as p < .0001.

Integrated microbiome–metabolomic analysis was performed using the M^2^IA platform.^44^ Microbial abundance counts were normalized by percentages, and metabolites were normalized by log transformation. Differential metabolites and microbes between groups were selected using univariate analysis. Spearman’s correlation coefficients were calculated between differential metabolites and microbes using a pairwise correlation analysis method with significance defined as p < .05 and R > 0.3 or < −0.3. Heatmaps were generated and visualized using a network plot. Spearman’s correlation coefficients were also calculated between differential microbes and clinical parameters including weight, fasting blood glucose, HbA1c, fasting serum insulin, HOMA-IR, low-density lipoproteins (LDL), HDL, triglycerides (TG), total cholesterol (TC), and CRP.

Supervised multivariable analysis was conducted using sPLS-DA to create score plots. Metabolic pathway enrichment analysis was performed on differential metabolites using univariate analysis. KEGG-based function of microbiome data was predicted using Tax4Fun2 following the linear discriminate analysis method.^45^ Overlapping pathways were identified, and interaction network plots were created demonstrating potential metabolites and microbes involved in these specific pathways.

## Supplementary Material

Supplemental Material

## Data Availability

The data that support the findings of this study are available on request from the corresponding author, JD. The data are not publicly available due to information that could compromise the privacy of research participants.
