# Supplementary material for: Roux-en-Y gastric bypass and sleeve gastrectomy induce substantial and persistent changes in microbial communities and metabolic pathways
Source: Gut Microbes. 2022 Mar 22;14(1):2050636. doi: 10.1080/19490976.2022.2050636 (PMC8942407; doi:10.1080/19490976.2022.2050636)
Supplement: Supplemental Material [file KGMI_A_2050636_SM0636.docx]

Supplementary Table 1. Clinical biochemistry results

| Blood test  mean (sd) | Non-operative controls  n = 25 | | | | Sleeve gastrectomy  n = 18 | | | | Roux-en-Y gastric bypass  n = 27 | | |
| --- | --- | --- | --- | --- | --- | --- | --- | --- | --- | --- | --- |
|  | 0 months | 3 months | 9 months | 0 months | | 3 months | 9 months | 0 months | | 3 months | 9 months |
| Hemoglobin (g/L) | 139.0 (9.5) | 137.6 (8.8) | 133.6 (32.6) | 134.6 (8.3) | | 135.9 (8.7) | 135.5 (8.2) | 140.3 (10.8) | | 137.8 (10.8) | 135.9 (10.5) |
| White blood cell (10^9^/L) | 6.8 (1.8) | 7.0 (1.7) | 6.5 (2.2) | 6.0 (1.9) | | 5.3 (1.0) | 5.0 (1.2) | 7.4 (2.0) | | 6.4 (1.9) | 5.9 (1.7) |
| Creatinine (µmol/L) | 68.9 (10.6) | 71.0 (11.7) | 68.9 (13.5) | 67.8 (8.9) | | 69.2 (10.0) | 69.5 (9.5) | 71.4 (15.1) | | 65.7 (12.9) | 65.6 (13.4) |
| ALT (U/L) | 24.2 (12.6) | 25.3 (14.4) | 25.4 (13.7) | 24.6 (9.1) | | 28.6 (15.7) | 24.8 (11.8) | 27.4 (14.1) | | 22.3 (8.2) | 25.3 (12.8) |
| ALP (U/L) | 85.0 (17.7) | 86.1 (21.9) | 89.3 (26.7) | 70.2 (17.) | | 78.2 (20.9) | 72.7 (14.1) | 77.7 (18.4) | | 86.2 (17.7) | 93.4 (19.3) |
| Bilirubin (µmol/L) | 9.0 (3.4) | 9.3 (3.7) | 10.1 (4.7) | 9.8 (4.1) | | 11.3 (4.7) | 9.5 (3.1) | 10.4 (5.5) | | 10.9 (4.6) | 10.2 (3.7) |
| Ferritin (µg/L) | 101.5 (84.4) | 89.4 (79.7) | 95.1 (54.5) | 74.5 (75.4) | | 91.8 (61.4) | 79.6 (53.0) | 103.8 (81.1) | | 72.7 (70.4) | 60 (48.9) |
| TSH (mU/L) | 2.13 (0.97) | 2.26 (1.04) | 2.00 (0.80) | 1.78 (1.16) | | 1.44 (0.87) | 1.81 (1.59) | 2.11 (1.01) | | 1.46 (0.84) | 1.65 (0.85) |
| Free T4 (pmol/L) | 14.5 (1.4) | 14.8 (1.5) | 14.5 (1.2) | 15.2 (2.8) | | 15.1 (3.5) | 14.7 (2.2) | 14.4 (1.9) | | 14.9 (2.2) | 13.5 (2.9) |
| Total cholesterol (mmol/L) | 4.4 (0.9) | 4.5 (0.9) | 4.2 (0.8) | 4.7 (0.8) | | 4.6 (0.9) | 5.1 (1.1) | 4.8 (1.0) | | 4.2 (1.1) | 4.4 (1.1) |
| Triglycerides (mmol/L) | 1.4 (0.6) | 1.4 (0.7) | 1.2 (0.6) | 1.4 (0.9) | | 1.2 (0.6) | 1.2 (0.5) | 1..5 (0.7) | | 1.1 (0.4) | 1.0 (0.4) |
| LDL (mmol/L) | 2.5 (0.8) | 2.5 (0.8) | 2.4 (0.7) | 2.7 (0.8) | | 2.7 (0.8) | 3.0 (1.0) | 2.8 (0.8) | | 2.5 (0.9) | 2.5 (0.8) |
| HDL (mmol/L) | 1.2 (0.3) | 1.3 (0.3) | 1.2 (0.3) | 1.4 (0.3) | | 1.4 (0.2) | 1.6 (0.3) | 1.3 (0.3) | | 1.2 (0.3) | 1.5 (0.4) |
| Non-HDL (mmol/L) | 3.2 (0.8) | 3.2 (0.8) | 3.0 (0.7) | 3.3 (0.8) | | 3.3 (0.9) | 3.5 (1.0) | 3.5 90.9) | | 3.0 (1.0) | 2.9 (1.0) |
| Fasting blood glucose (mmol/L) | 5.4 (0.6) | 5.5 (0.9) | 5.4 (0.9) | 5.4 (1.1) | | 4.9 (0.4) | 4.8 (0.3) | 5.5 (0.8) | | 5.1 (0.4) | 5.0 (0.6) |
| Hemoglobin A1c (%) | 5.6 (0.3) | 5.7 (0.4) | 5.6 (0.5) | 5.8 (0.5) | | 5.5 (0.3) | 5.5 (0.2) | 5.7 (0.8) | | 5.4 (0.4) | 5.3 (0.4) |
| Insulin (pmol/L) | 198.6 (167.3) | 191.6 (150.0) | 159.8 (97.2) | 113.3 (50.5) | | 66.5 (47.9) | 50.4 (18.8) | 115.8 (51.3) | | 64.4 (23.4) | 44.4 (14.7) |

ALT, alanine aminotransferase; ALP, alkaline phosphatase; TSH, thyroid stimulating hormone; LDL, low-density lipoproteins; HDL, high-density lipoproteins

Supplementary Table 2. C-reactive protein, lipopolysaccharide, and inflammatory cytokines

| Blood test  mean (sd) | Non-operative controls  n = 25 | | | Sleeve gastrectomy  n = 18 | | | Roux-en-Y gastric bypass  n = 27 | | |
| --- | --- | --- | --- | --- | --- | --- | --- | --- | --- |
|  | 0 months | 3 months | 9 months | 0 months | 3 months | 9 months | 0 months | 3 months | 9 months |
| C-reactive protein (mg/L) | 11.2 (9.4) | 11.1 (7.7) | 16.6 (25.5) | 6.0 (6.1) | 4.4 (5.9) | 3.5 (4.6) | 9.5 (6.8) | 4.0 (5.4) | 2.5 (3.9) |
| Lipopolysaccharide (EU/mL) | 2.4 (2.1) | 2.0 (1.5) | 2.9 (4.0) | 1.6 (0.7) | 1.4 (0.7) | 1.7 (1.0) | 1.7 (1.2) | 1.5 (0.7) | 1.9 (1.2) |
| IL-1β (pg/mL) | 36.2 (104.1) | 44.8 (106.8) | 52.8 (151.9) | 72.0 (179.4) | 81.3 (215.1) | 112.4 (224.2) | 94.6 (202.0) | 143.0 (245.1) | 118.6 (221.9) |
| IL-6 (pg/mL) | 41.1 (88.2) | 51.4 (103.4) | 43.8 (86.4) | 87.1 (191.4) | 113.4 (214.5) | 134.7 (242.8) | 83.6 (184.2) | 160.1 (300.2) | 96.6 (186.1) |
| IL-8 (pg/mL) | 25.7 (56.5) | 29.5 (60.3) | 32.6 (72.1) | 45.7 (85.5) | 57.8 (106.9) | 105.4 (258.8) | 25.5 (46.7) | 86.6 (166.3) | 48.6 (84.) |
| IL-10 (pg/mL) | 204.0 (415.7) | 258.8 (511.5) | 261.4 (552.4) | 403.5 (718.1) | 486.3 (751.0) | 632.6 (930.5) | 387.3 (680.7) | 504.5 (802.8) | 577.5 (917.3) |
| TNF-α (pg/mL) | 6.0 (1.2) | 6.2 (1.4) | 5.9 (1.6) | 6.2 (1.5) | 7.3 (4.7) | 7.3 (4.4) | 21.5 (70.1) | 44.8 (134.1) | 24.8 (79.1) |

#

Supplementary Figure 1. STROBE flow chart for observational studies

Supplementary Figure 2. Body mass index. CTRL, non-operative control; SG, sleeve gastrectomy; RYGB, Roux-en-Y gastric bypass

Supplementary Figure 3. Lipid panel. CTRL, non-operative control (n=25); SG, sleeve gastrectomy (n=18); RYGB, Roux-en-Y gastric bypass (n=27); LDL, low-density lipoprotein; HDL, high-density lipoprotein. Error bars on figures represent standard error of the means and asterisks represent statistical significance with * as p<0.05, ** as p<0.01, *** as p<0.001, **** as p<0.0001.

Supplementary Figure 4. Metabolic parameters. CTRL, non-operative control (n=25); SG, sleeve gastrectomy (n=18); RYGB, Roux-en-Y gastric bypass (n=27); HbA1c, hemoglobin A1c; HOMA-IR, homeostatic model for the assessment of insulin resistance. Error bars on figures represent standard error of the means and asterisks represent statistical significance with * as p<0.05, ** as p<0.01, *** as p<0.001, **** as p<0.0001.

Supplementary Figure 5. Inflammatory markers. CTRL, non-operative control (n=25); SG, sleeve gastrectomy (n=18); RYGB, Roux-en-Y gastric bypass (n=27). Error bars on figures represent standard error of the means and asterisks represent statistical significance with * as p<0.05, ** as p<0.01, *** as p<0.001, **** as p<0.0001.

Supplementary Figure 6. Interleukins. CTRL, non-operative control; SG, sleeve gastrectomy; RYGB, Roux-en-Y gastric bypass.


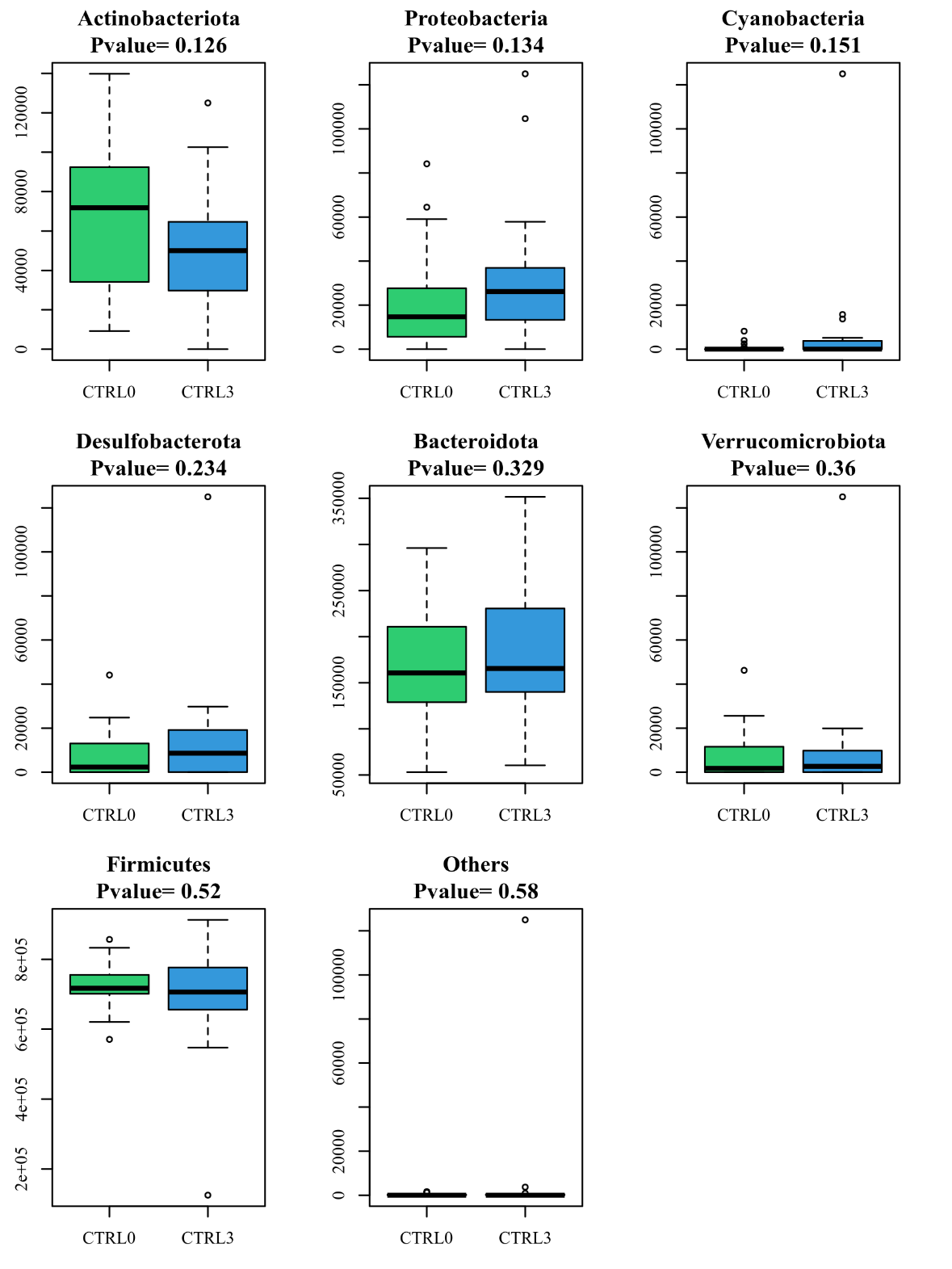


Supplementary Figure 7. Differential microbial taxa on univariate analysis at the phylum level between baseline and 3 months for non-operative control. Raw, non-corrected p-values are displayed.


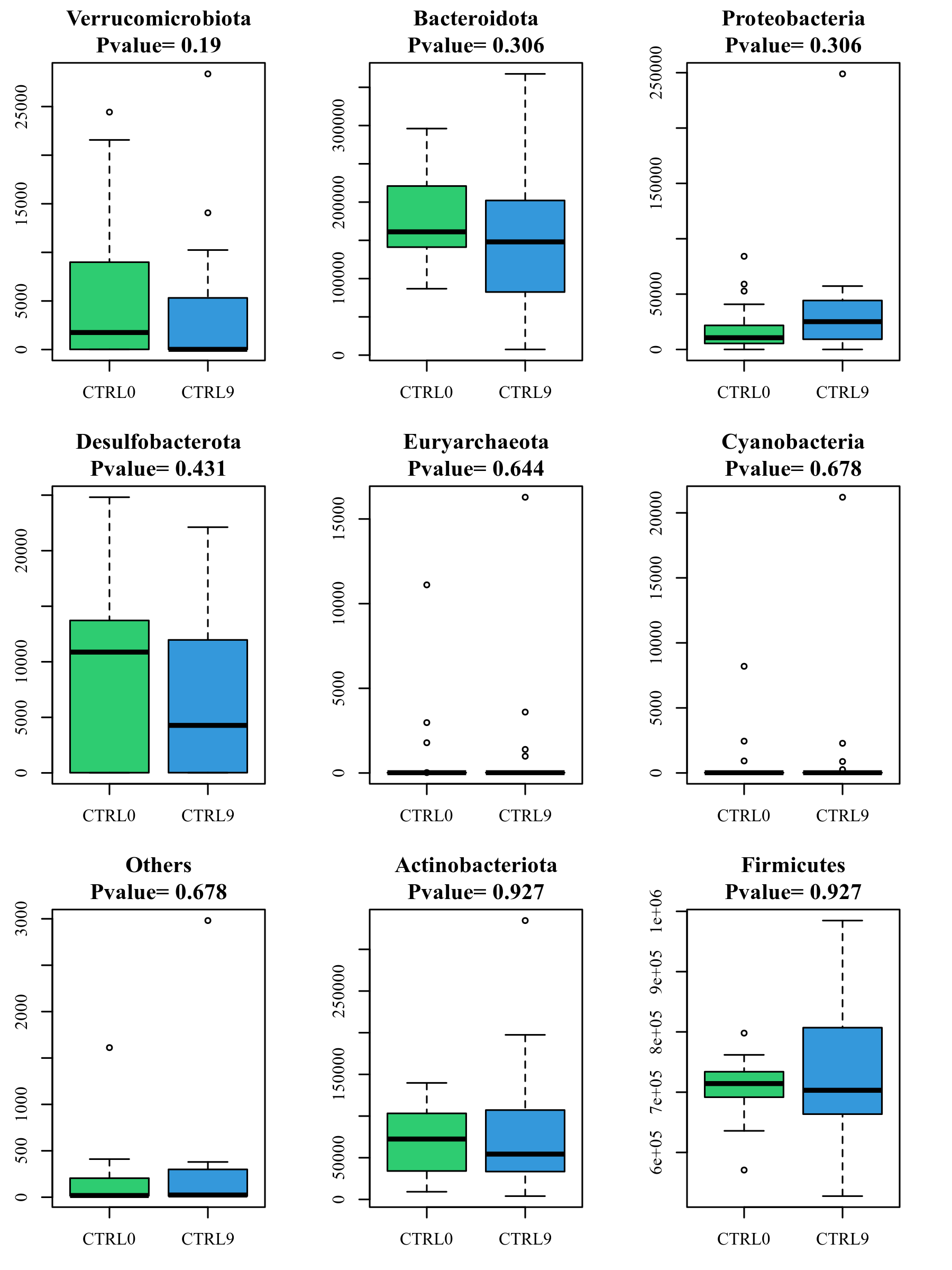


Supplementary Figure 8. Differential microbial taxa on univariate analysis at the phylum level between baseline and 9 months for non-operative control. Raw, non-corrected p-values are displayed.


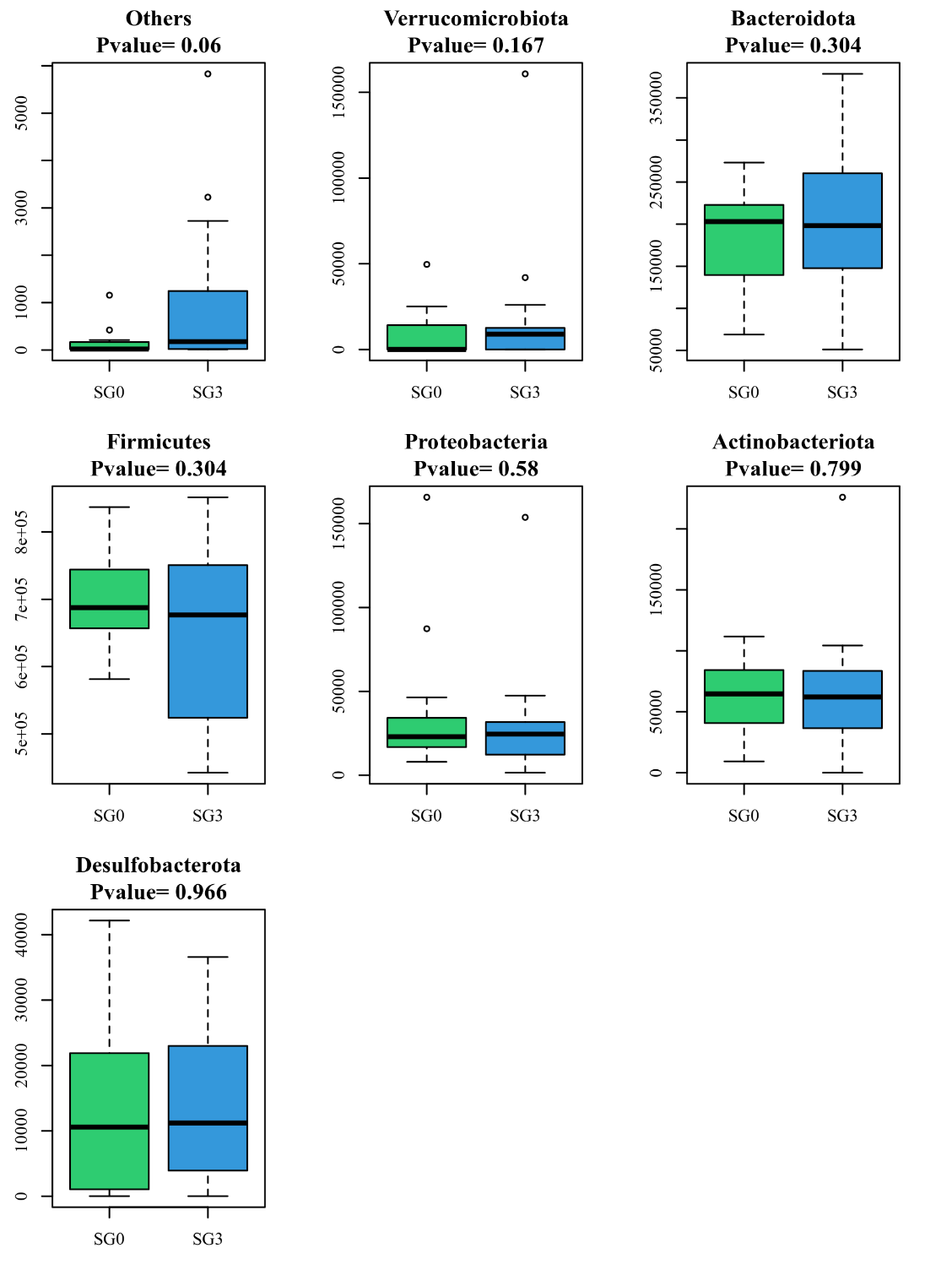


Supplementary Figure 9. Differential microbial taxa on univariate analysis at the phylum level between baseline and 3 months for sleeve gastrectomy. Raw, non-corrected p-values are displayed.


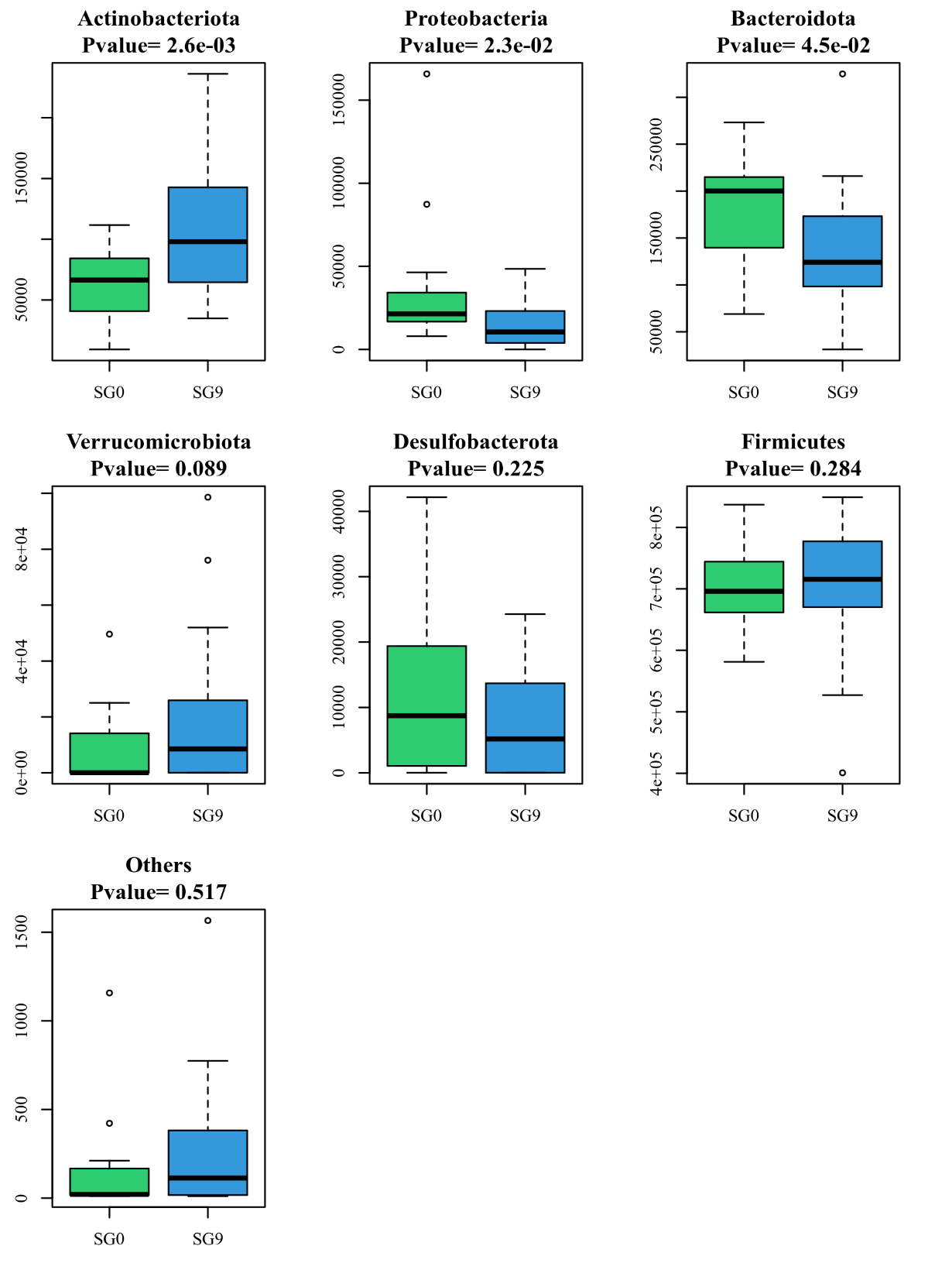


Supplementary Figure 10. Differential microbial taxa on univariate analysis at the phylum level between baseline and 9 months for sleeve gastrectomy. Raw, non-corrected p-values are displayed.


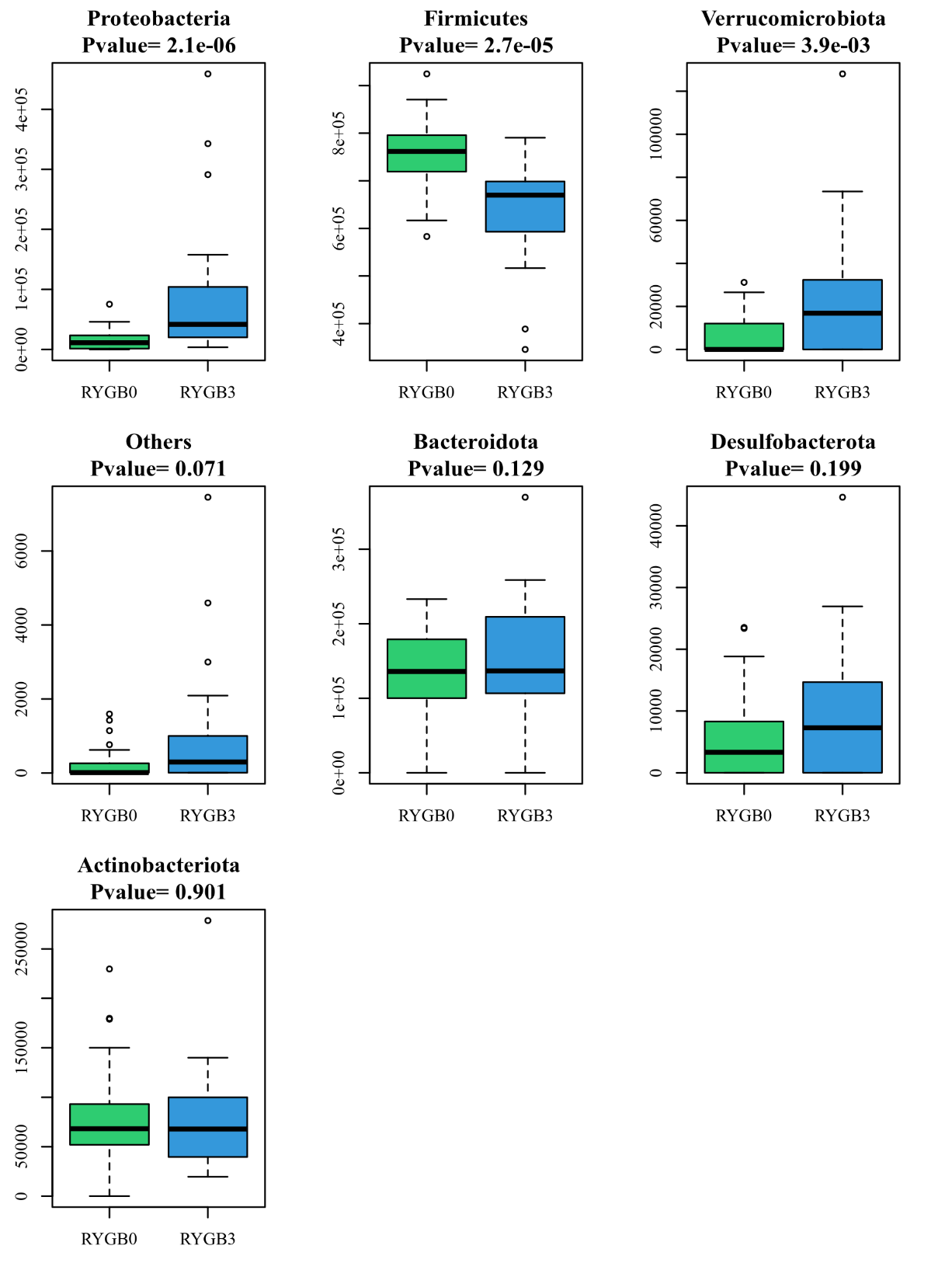


Supplementary Figure 11. Differential microbial taxa on univariate analysis at the phylum level between baseline and 3 months for Roux-en-Y gastric bypass. Raw, non-corrected p-values are displayed.


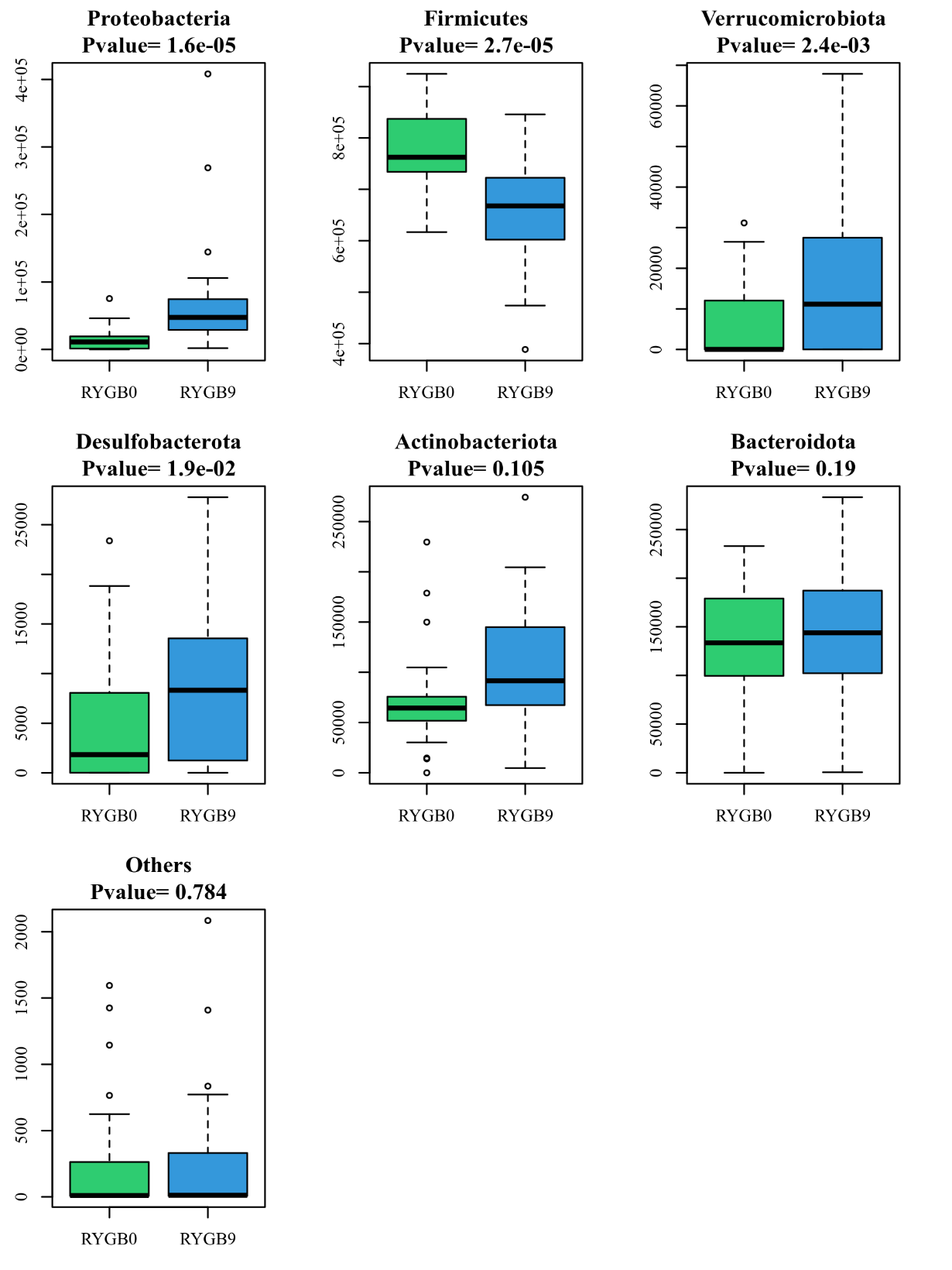


Supplementary Figure 12. Differential microbial taxa on univariate analysis at the phylum level between baseline and 9 months for Roux-en-Y gastric bypass. Raw, non-corrected p-values are displayed.


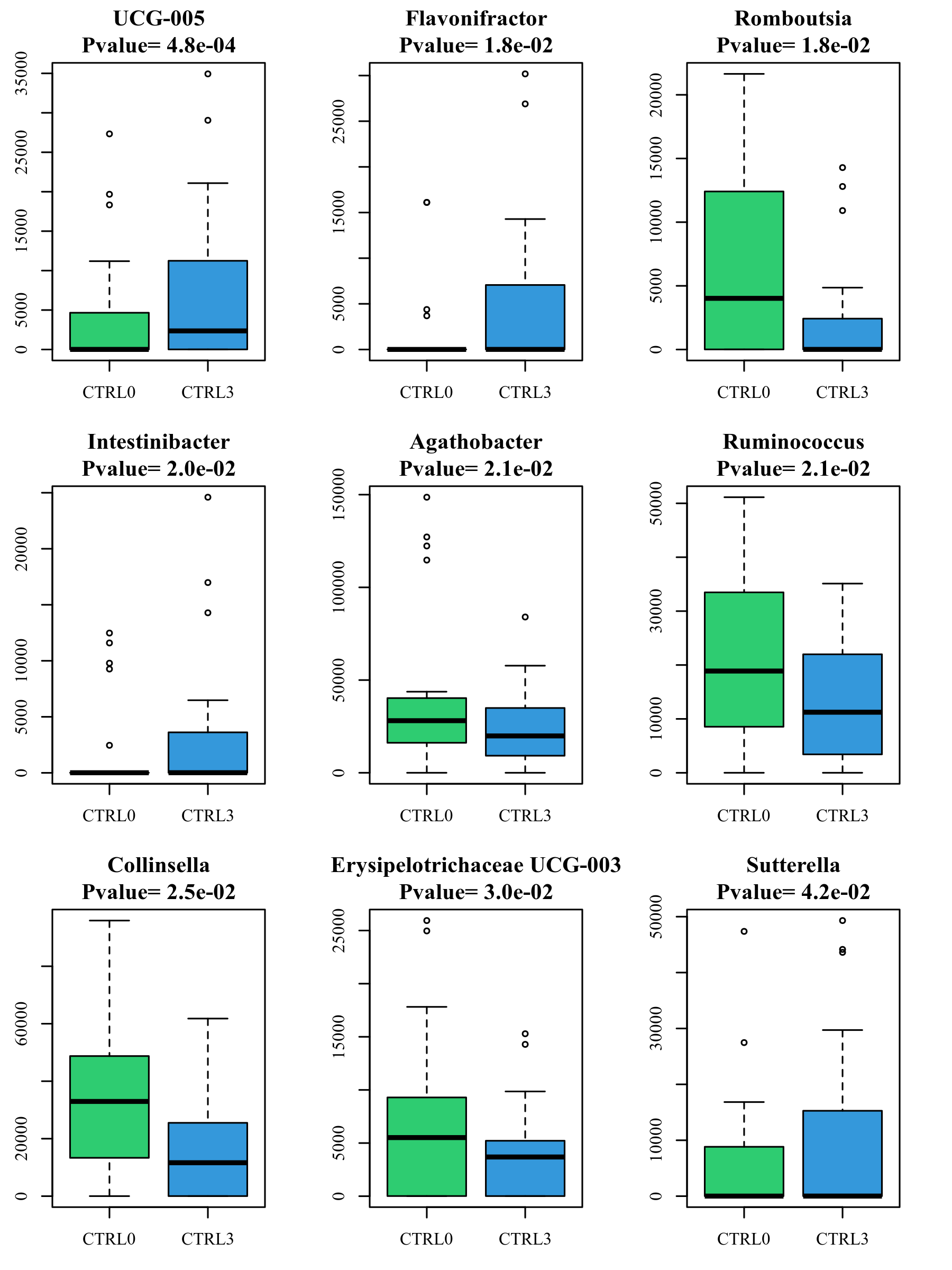


Supplementary Figure 13. Differential microbial taxa on univariate analysis at the genus level between baseline and 3 months for non-operative control. Raw, non-corrected p-values are displayed.


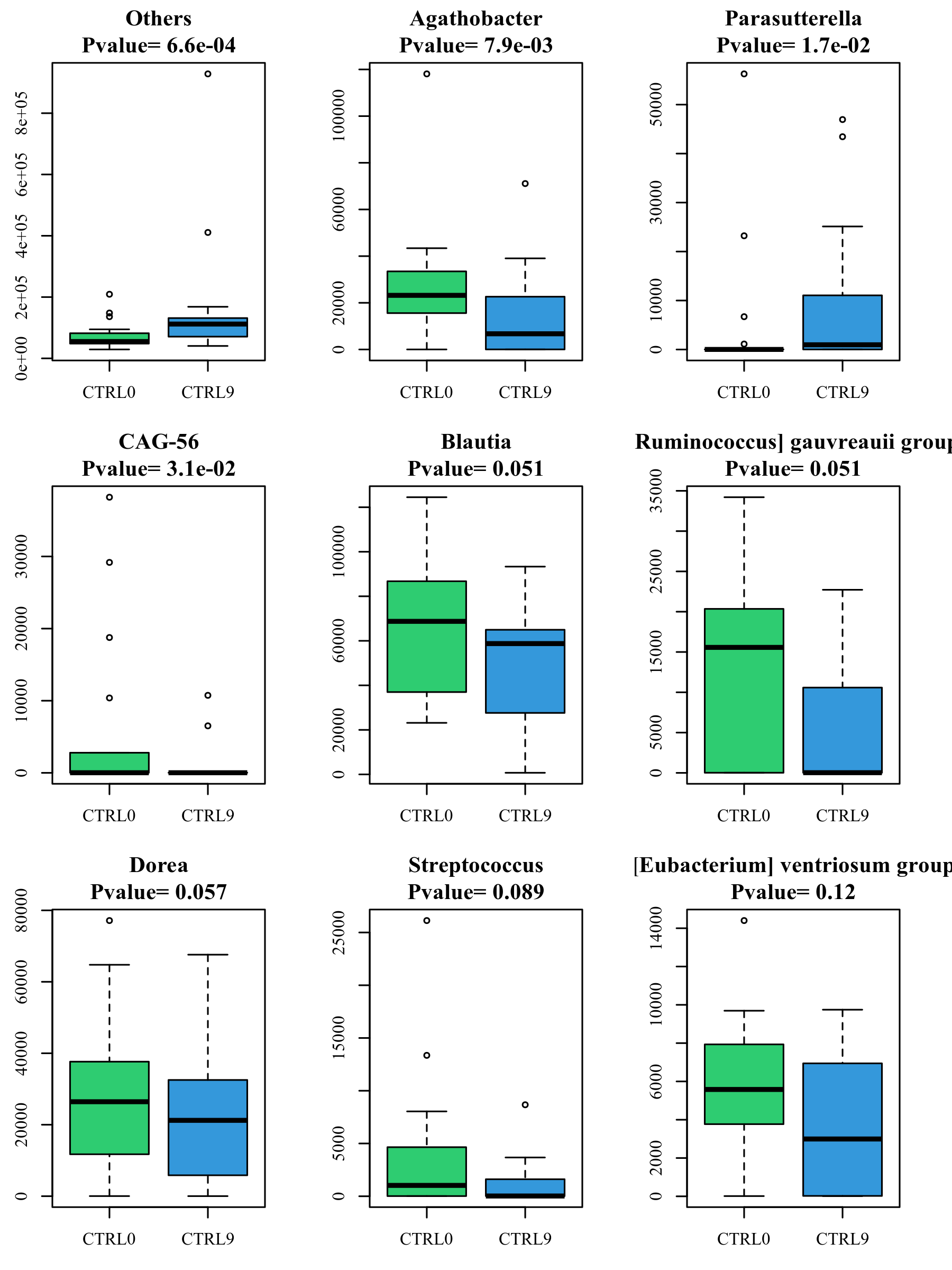


Supplementary Figure 14. Differential microbial taxa on univariate analysis at the genus level between baseline and 9 months for non-operative control. Raw, non-corrected p-values are displayed.


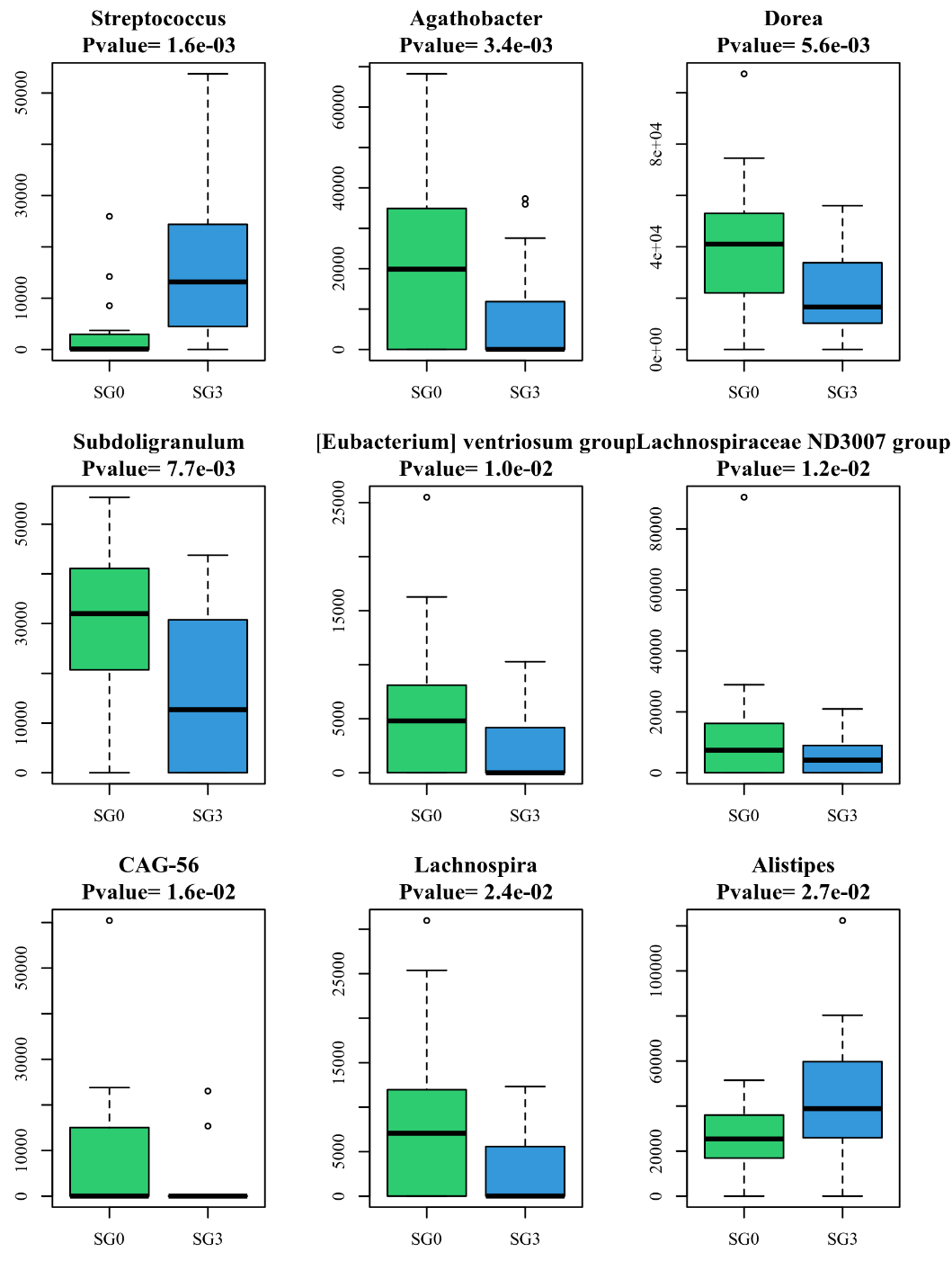


Supplementary Figure 15. Differential microbial taxa on univariate analysis at the genus level between baseline and 3 months for sleeve gastrectomy. Raw, non-corrected p-values are displayed.


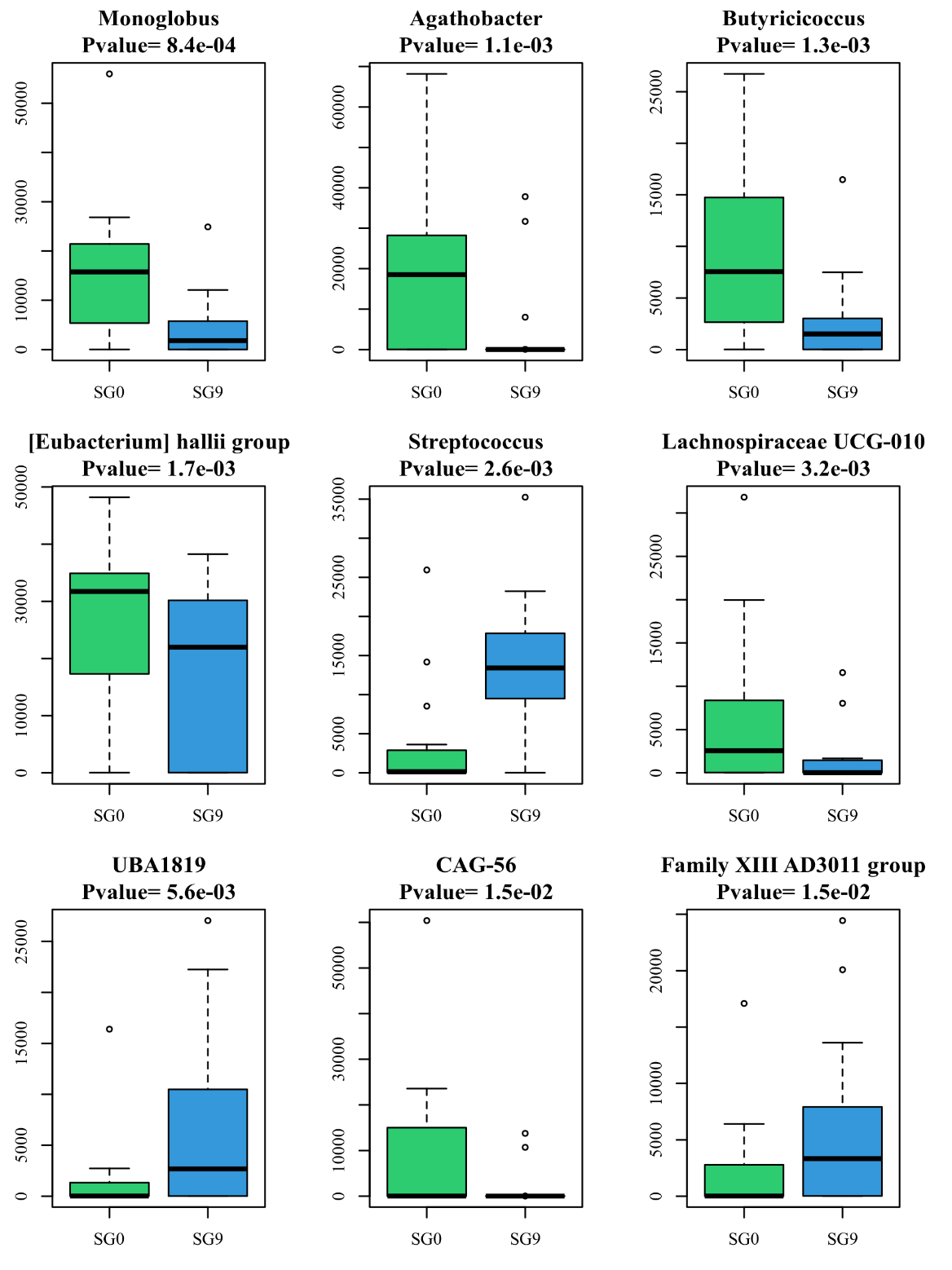


Supplementary Figure 16. Differential microbial taxa on univariate analysis at the genus level between baseline and 9 months for sleeve gastrectomy. Raw, non-corrected p-values are displayed.


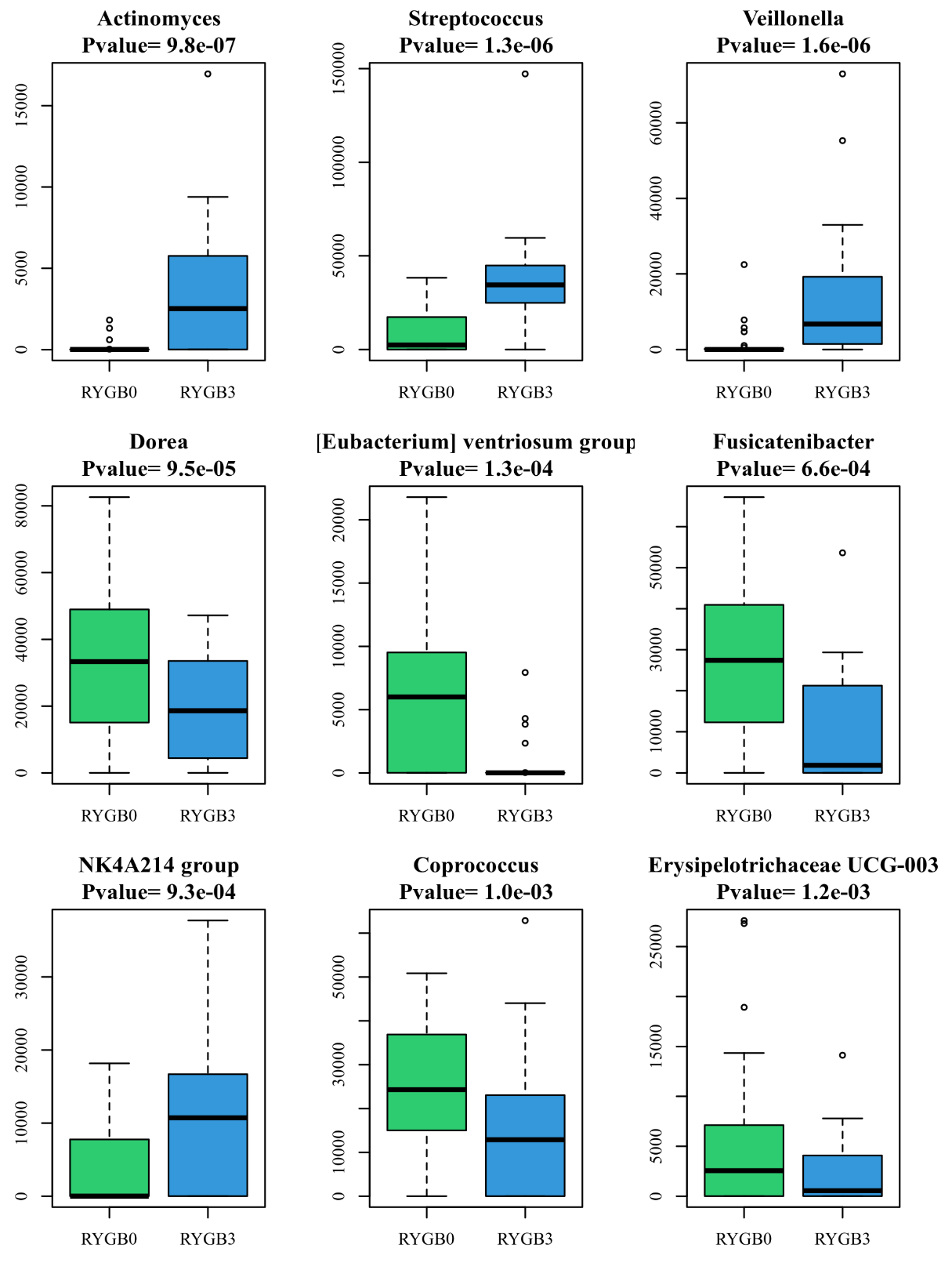


Supplementary Figure 17. Differential microbial taxa on univariate analysis at the genus level between baseline and 3 months for Roux-en-Y gastric bypass. Raw, non-corrected p-values are displayed.


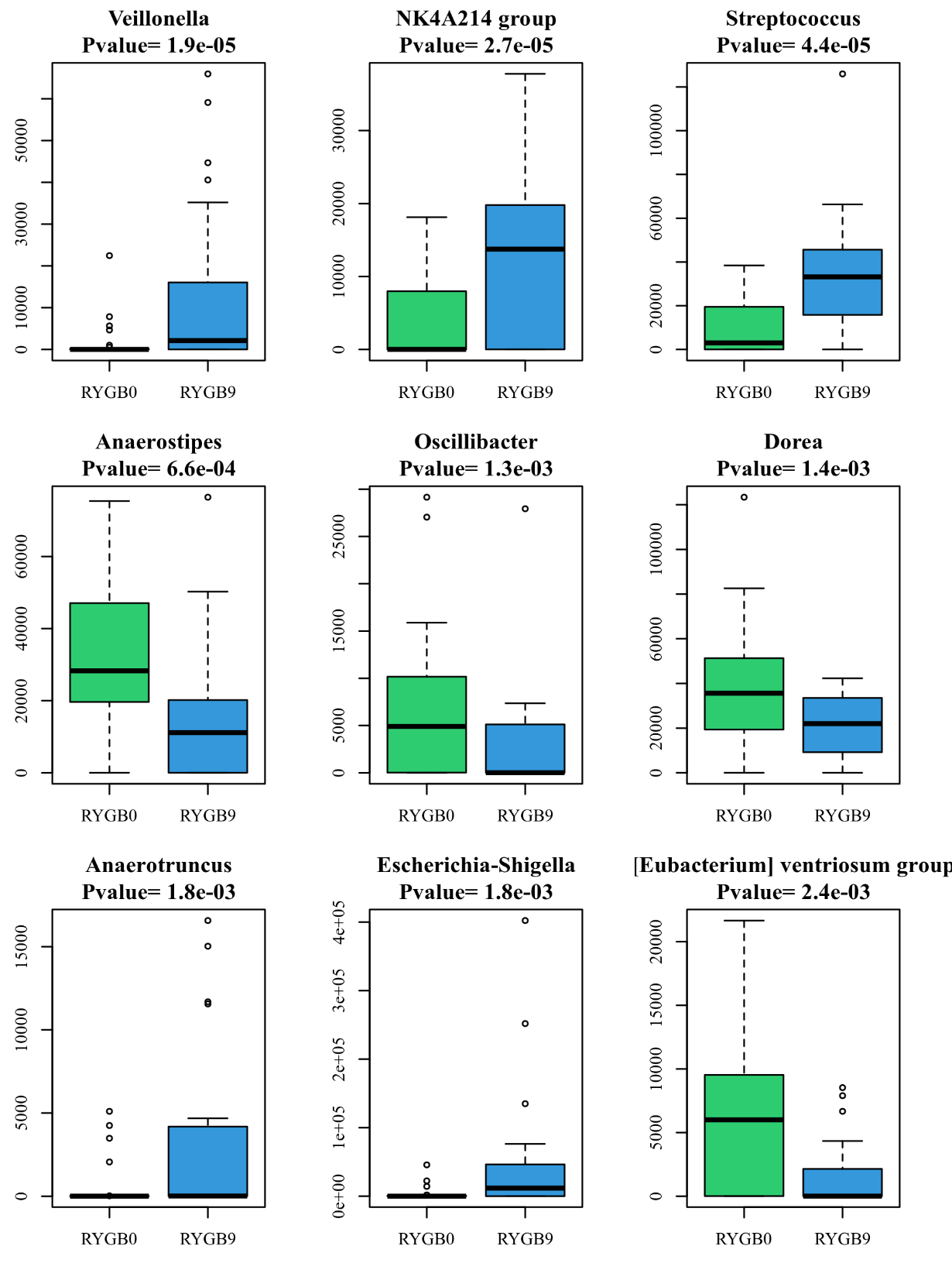


Supplementary Figure 18. Differential microbial taxa on univariate analysis at the genus level between baseline and 9 months for Roux-en-Y gastric bypass. Raw, non-corrected p-values are displayed.


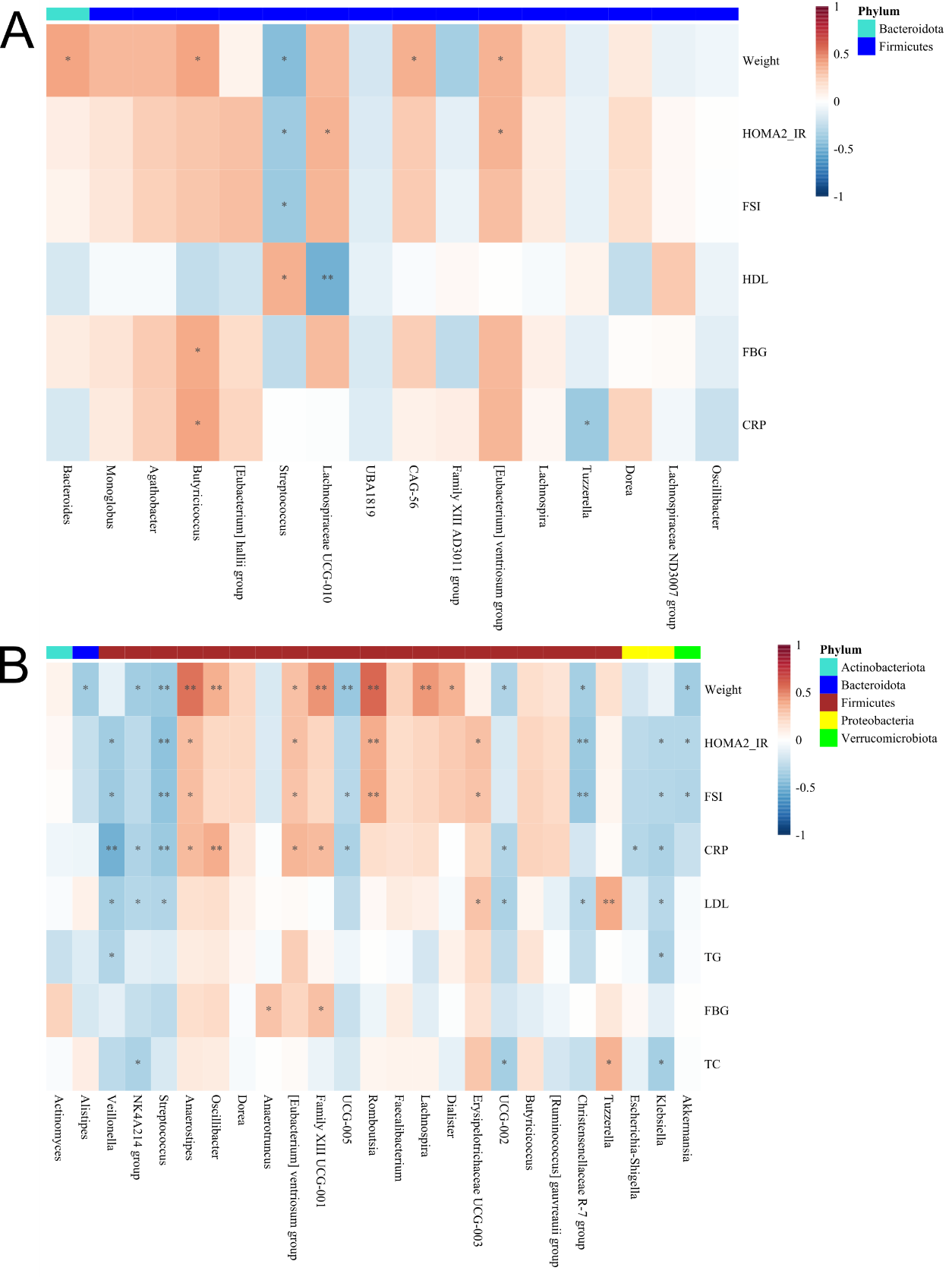


Supplementary Figure 19. Heatmaps demonstrating Spearman correlations between differential microbial genera and clinical parameters at 9 months compared to baseline for (A) sleeve gastrectomy and (B) Roux-en-Y gastric bypass. FBG, fasting blood glucose, FSI; fasting serum insulin; HOMA2-IR, Homeostasis model for the assessment of insulin resistance; LDL low-density lipoprotein; HDL, high-density lipoprotein; TG, triglyceride; TC, total cholesterol; CRP, C-reactive protein.


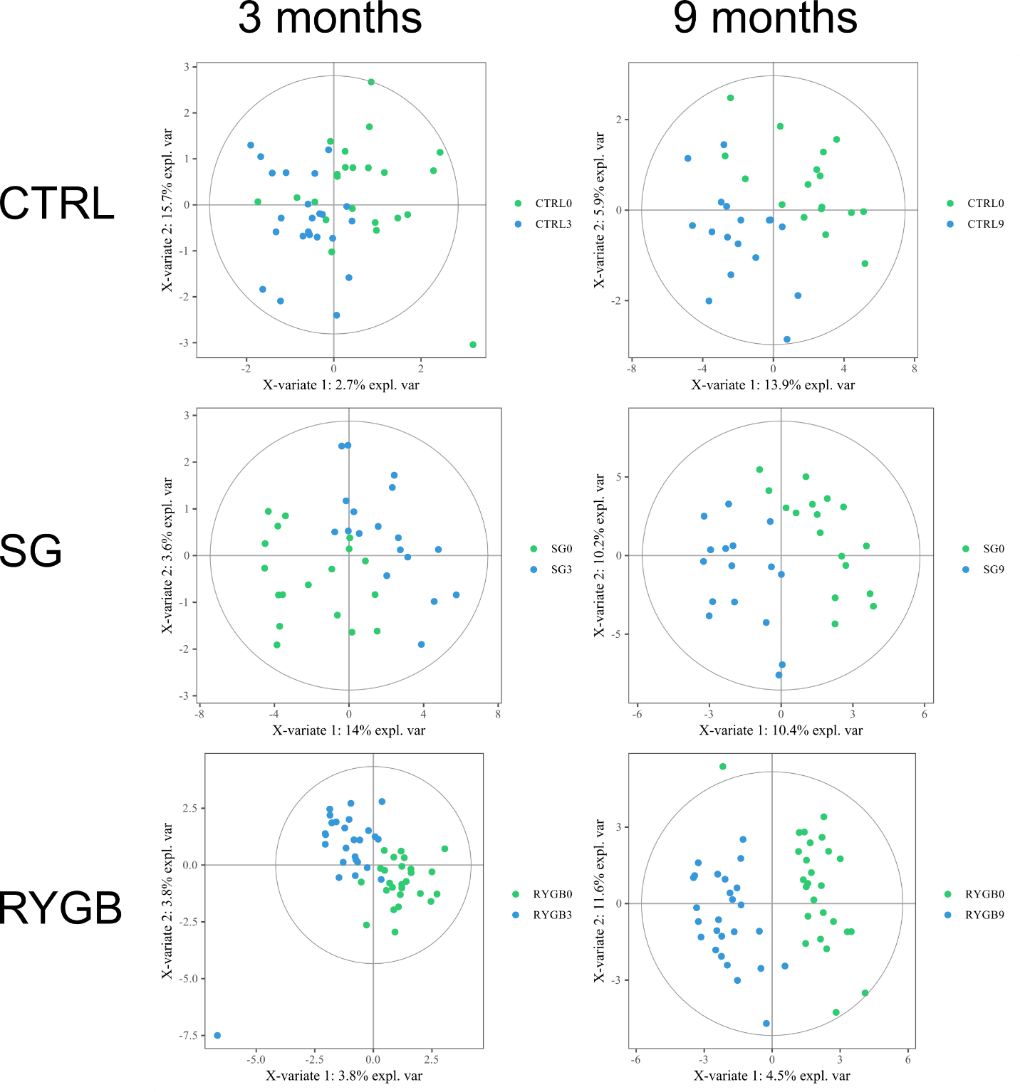


Supplementary Figure 20. Sparse partial least squares discriminant analysis score plots including microbial and metabolomic variables for non-operative control (CTRL), sleeve gastrectomy (SG) and Roux-en-Y gastric bypass cohorts (RYGB) at 3 and 9 months compared to baseline.


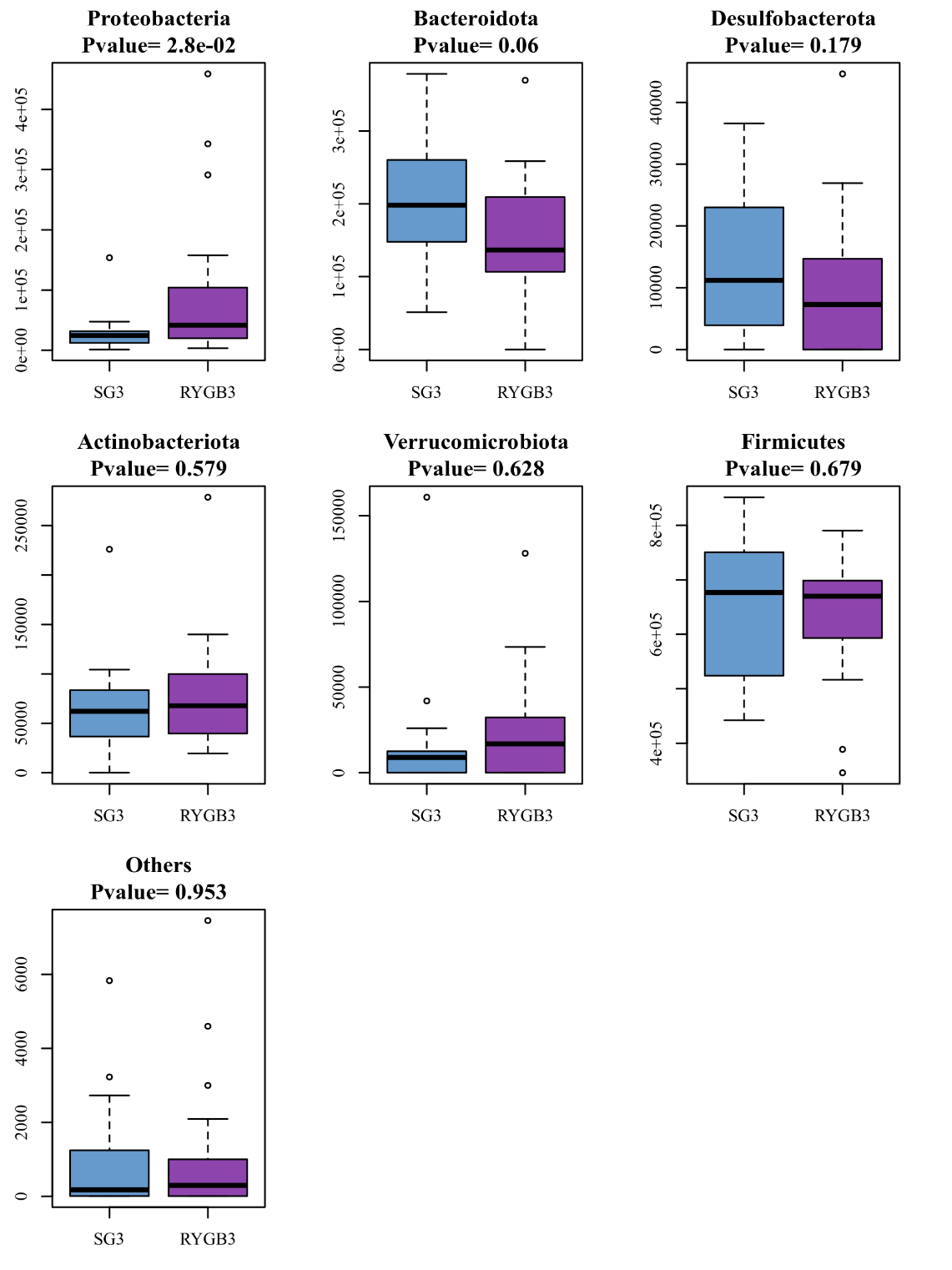


Supplementary Figure 21. Differential microbial taxa on univariate analysis at the phylum level between sleeve gastrectomy and Roux-en-Y gastric bypass at 3 months. Raw, non-corrected p-values are displayed.


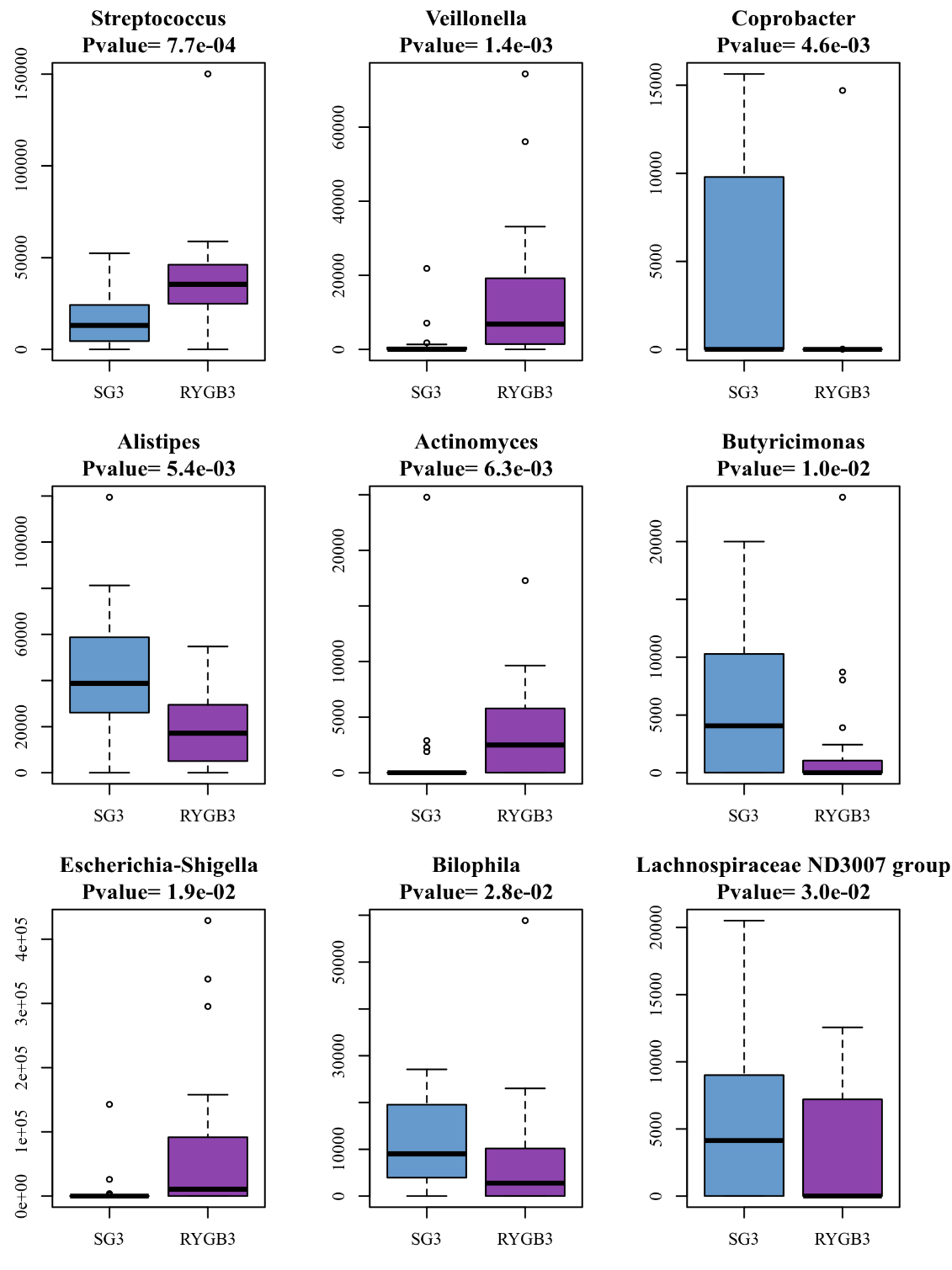


Supplementary Figure 22. Differential microbial taxa on univariate analysis at the genus level between sleeve gastrectomy and Roux-en-Y gastric bypass at 9 months. Raw, non-corrected p-values are displayed.


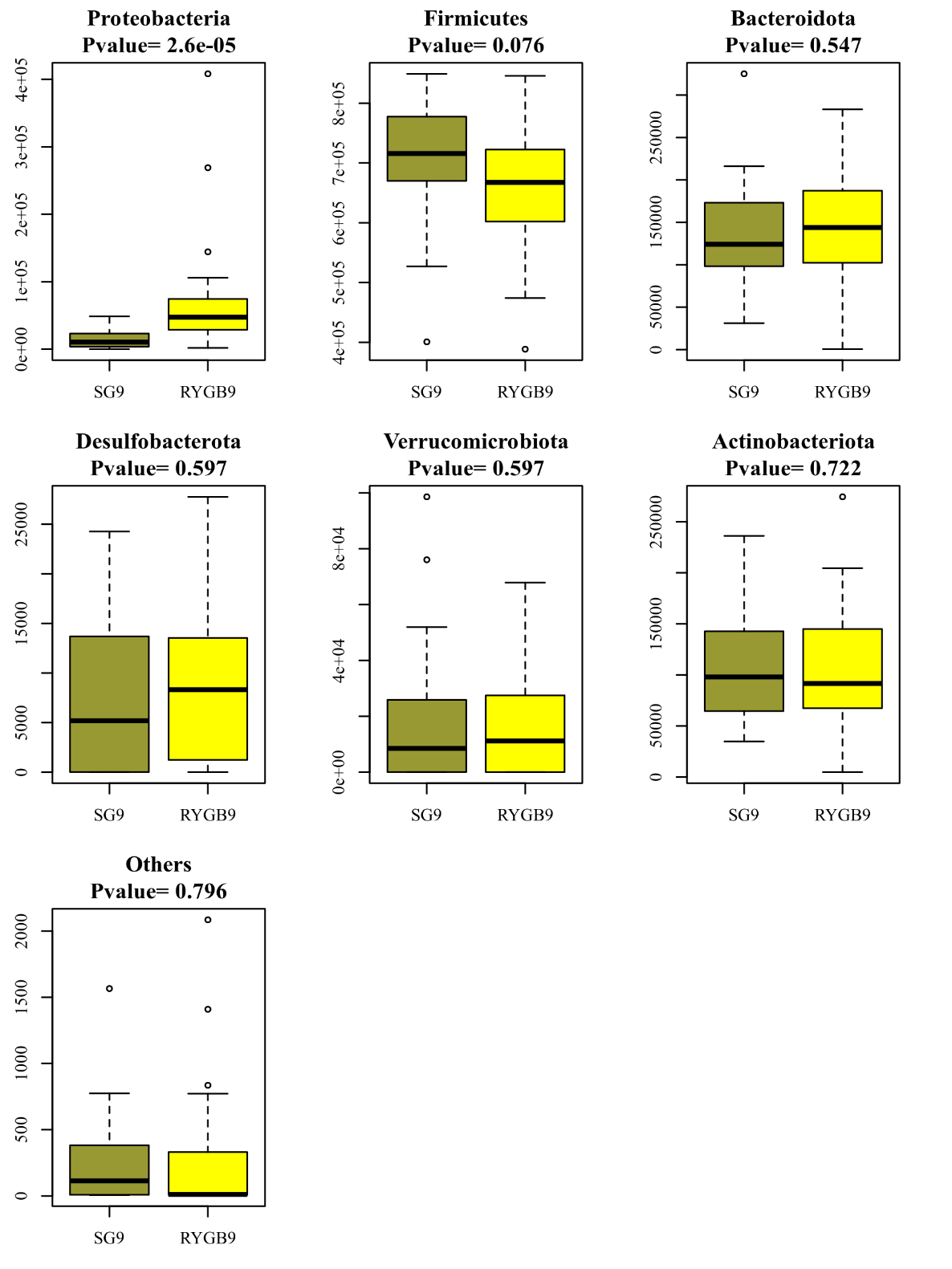


Supplementary Figure 23. Differential microbial taxa on univariate analysis at the phylum level between sleeve gastrectomy and Roux-en-Y gastric bypass at 9 months. Raw, non-corrected p-values are displayed.


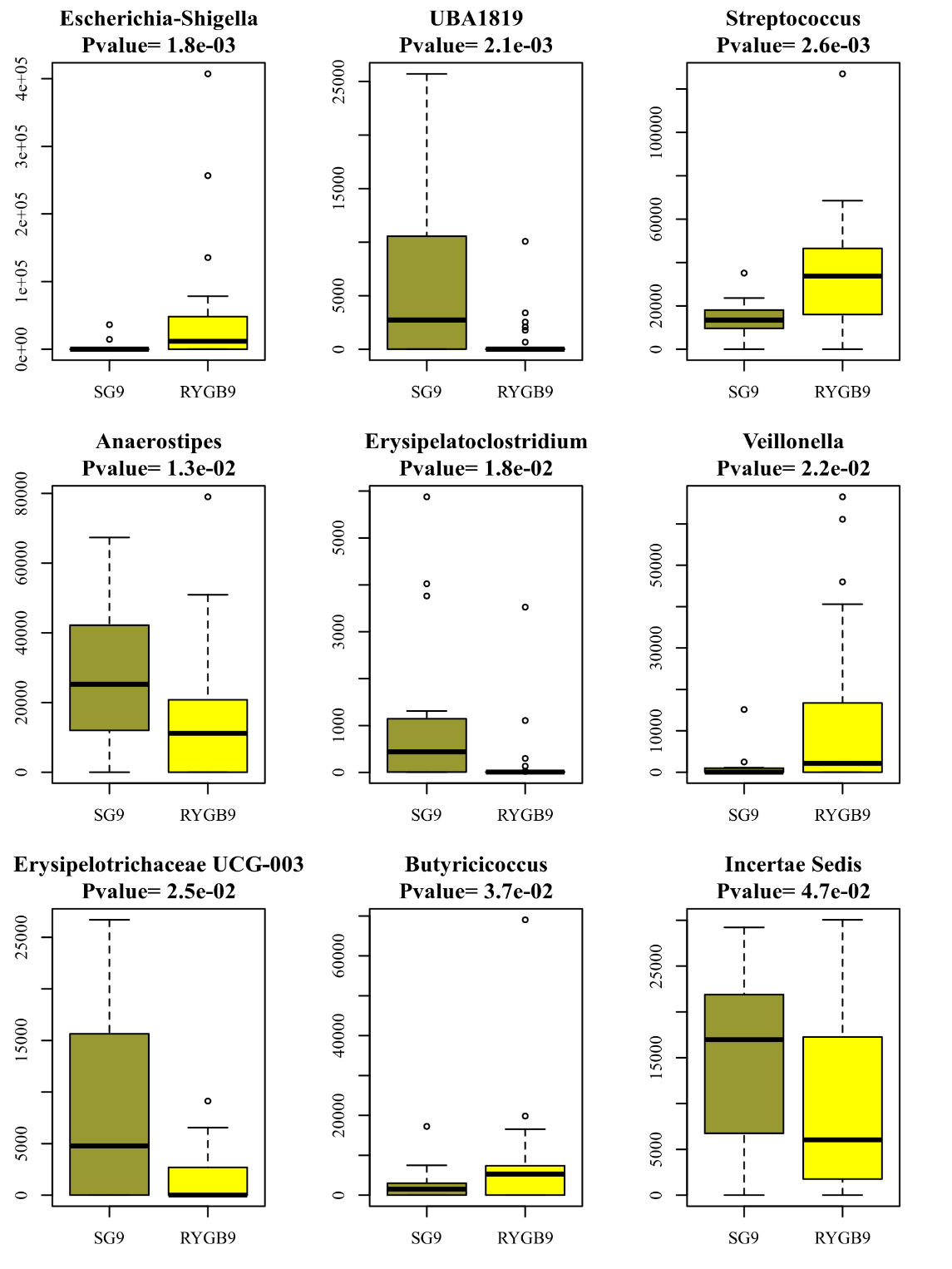


Supplementary Figure 24. Differential microbial taxa on univariate analysis at the genus level between sleeve gastrectomy and Roux-en-Y gastric bypass at 9 months. Raw, non-corrected p-values are displayed.


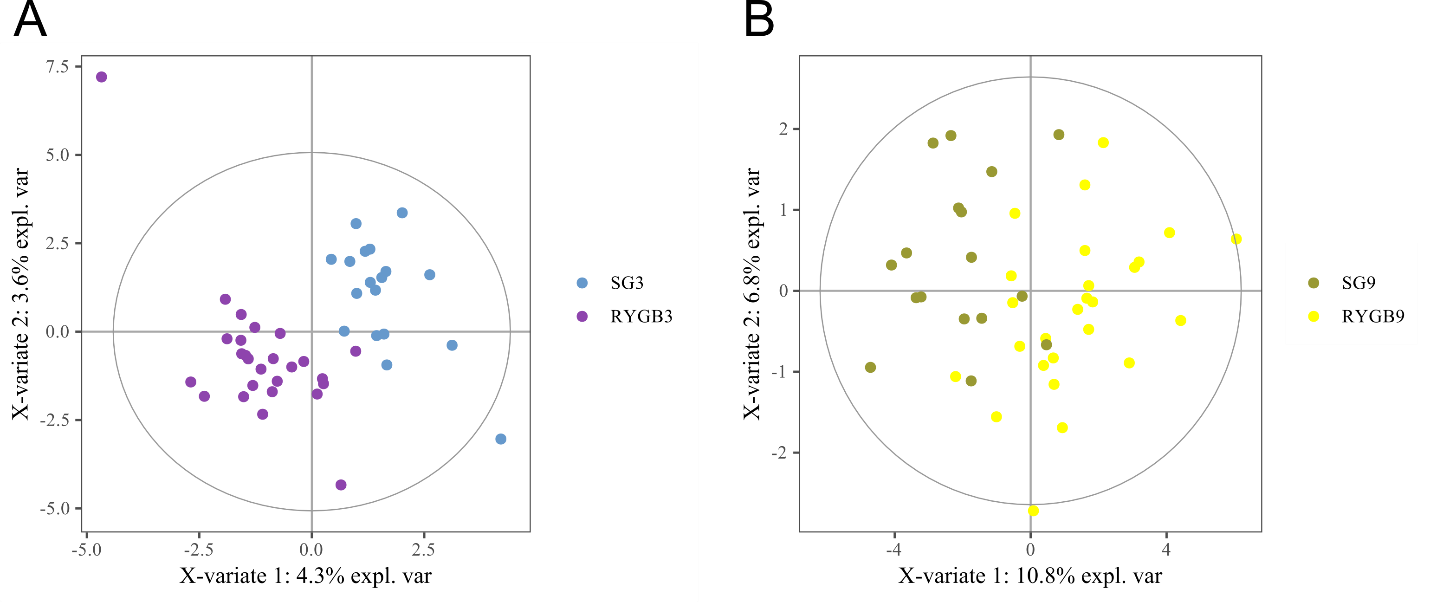


Supplementary Figure 25. Sparse partial least squares discriminant analysis score plots including microbial and metabolomic variables between sleeve gastrectomy and Roux-en-Y gastric bypass cohorts at (A) 3 months and (B) 9 months.


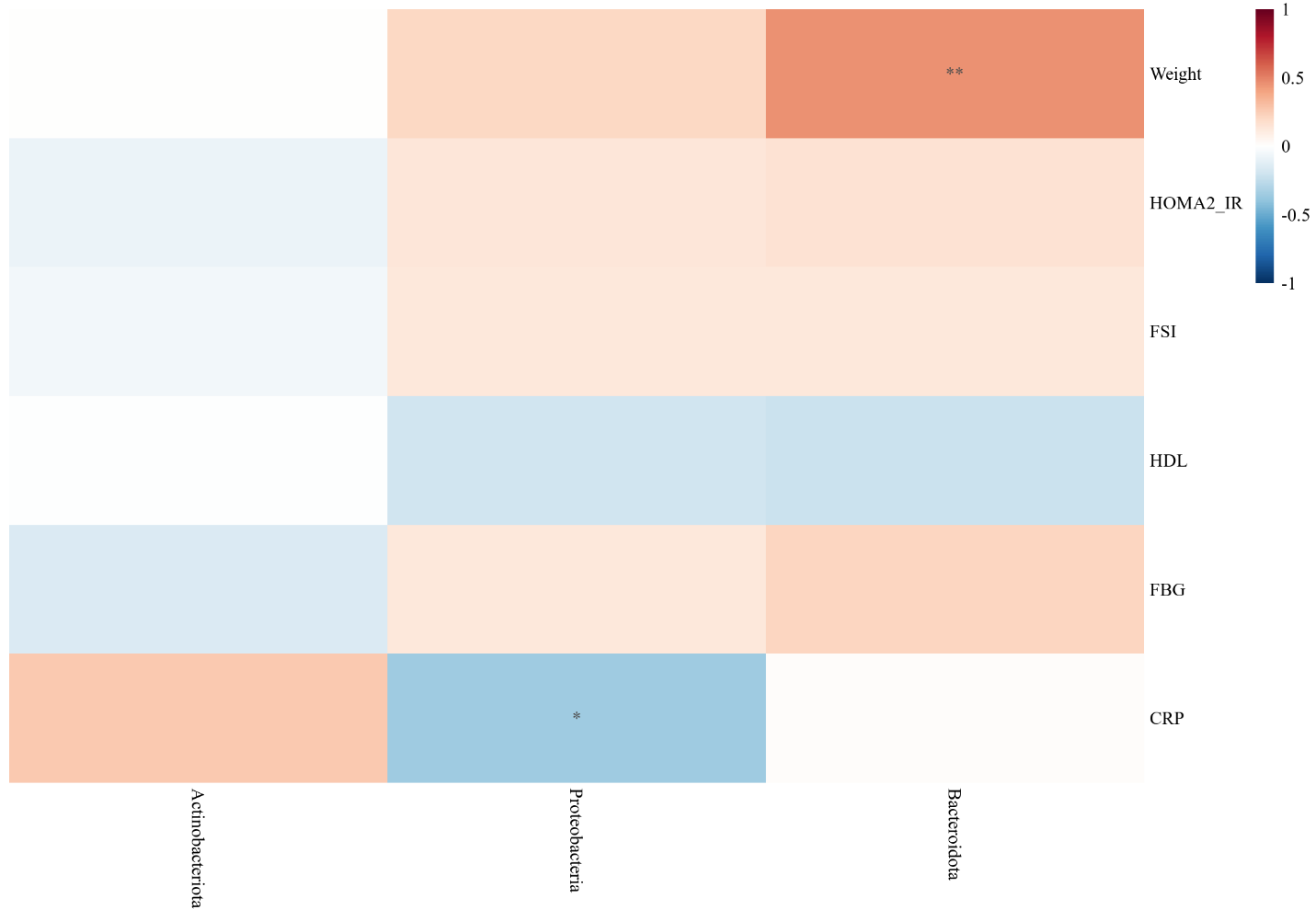


Supplementary Figure 26. Heatmap demonstrating Spearman correlations between differential microbial phlya and clinical parameters at 9 months compared to baseline for sleeve gastrectomy. FBG, fasting blood glucose, FSI; fasting serum insulin; HOMA2-IR, Homeostasis model for the assessment of insulin resistance; LDL low-density lipoprotein; HDL, high-density lipoprotein; TG, triglyceride; TC, total cholesterol; CRP, C-reactive protein.


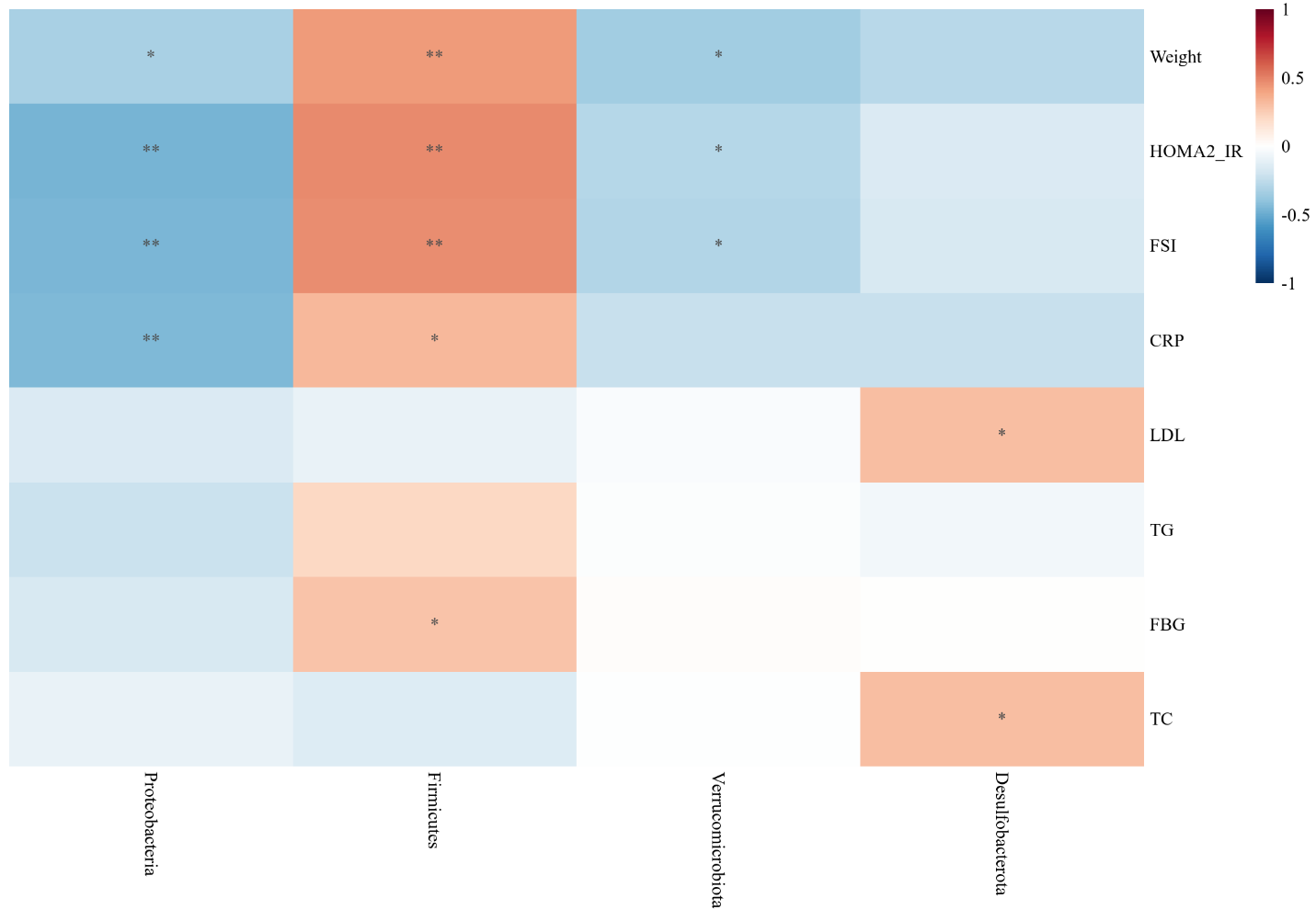


Supplementary Figure 27. Heatmap demonstrating Spearman correlations between differential microbial phlya and clinical parameters at 9 months compared to baseline for Roux-en-Y gastric bypass. FBG, fasting blood glucose, FSI; fasting serum insulin; HOMA2-IR, Homeostasis model for the assessment of insulin resistance; LDL low-density lipoprotein; HDL, high-density lipoprotein; TG, triglyceride; TC, total cholesterol; CRP, C-reactive protein.


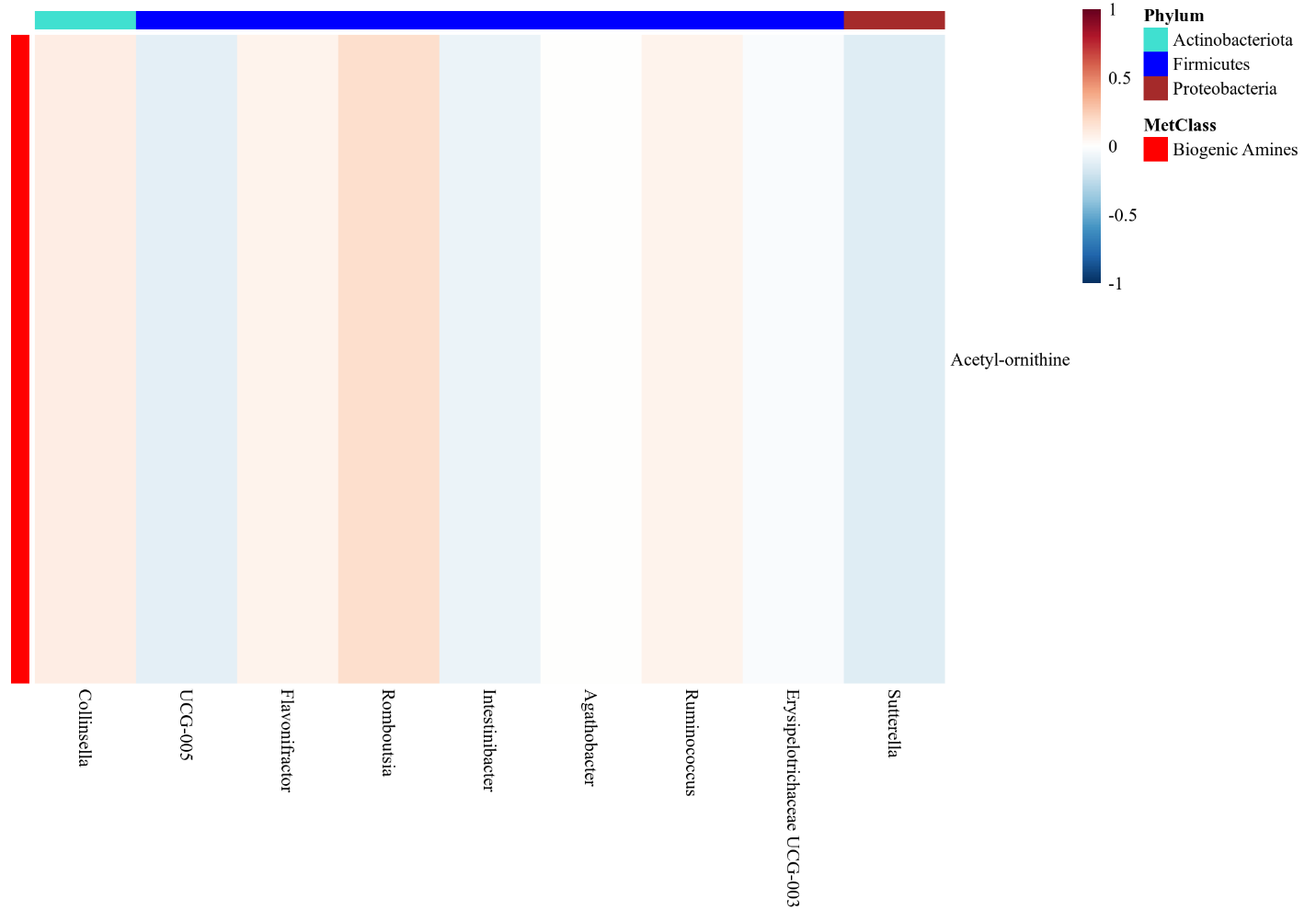


Supplementary Figure 28. Heatmap of Spearman correlations between differential microbes and metabolites at 3 months compared to baseline for non-operative control.


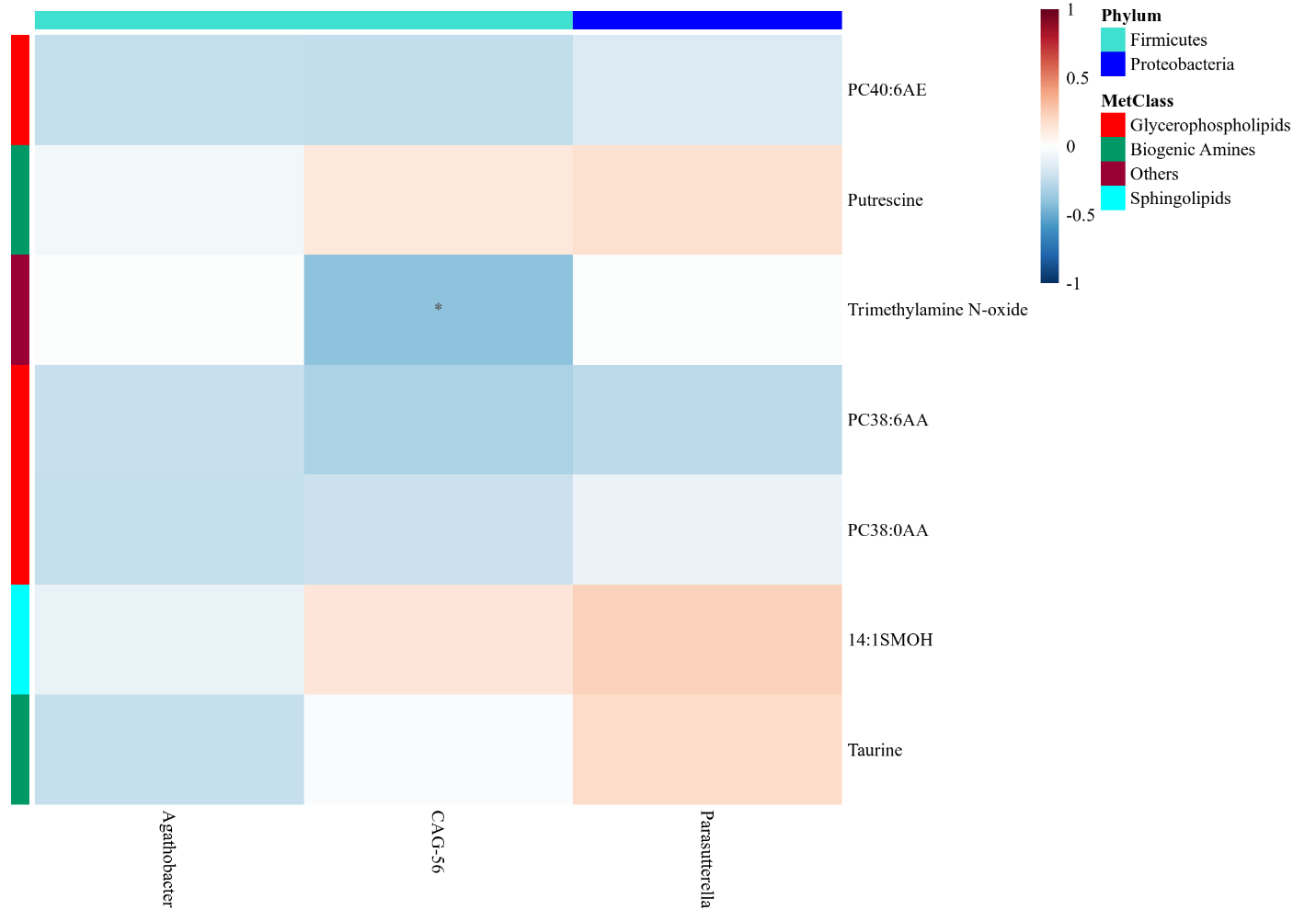


Supplementary Figure 29. Heatmap of Spearman correlations between differential microbes and metabolites at 9 months compared to baseline for non-operative control.


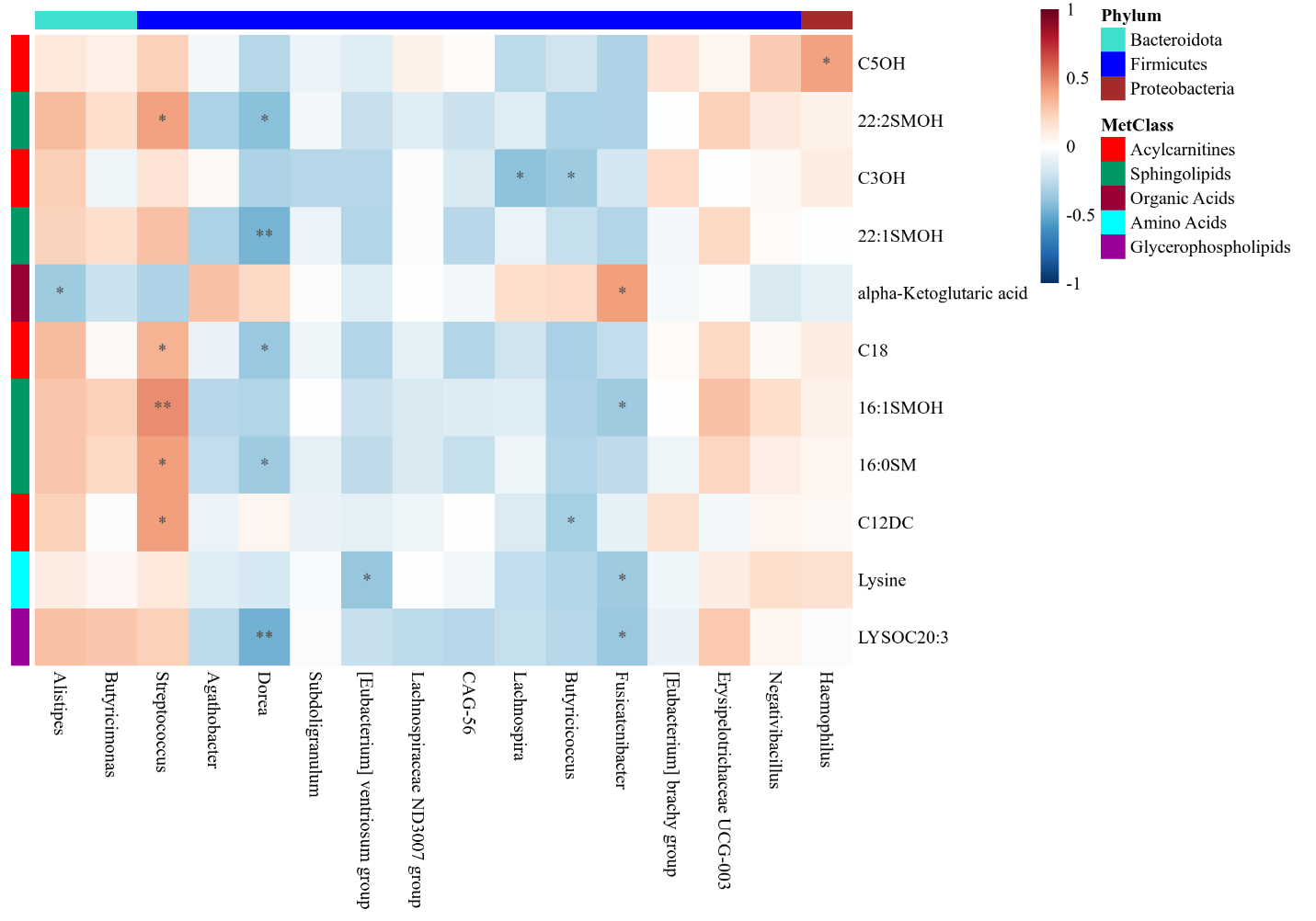


Supplementary Figure 30. Heatmap of Spearman correlations between differential microbes and metabolites at 3 months compared to baseline for sleeve gastrectomy.


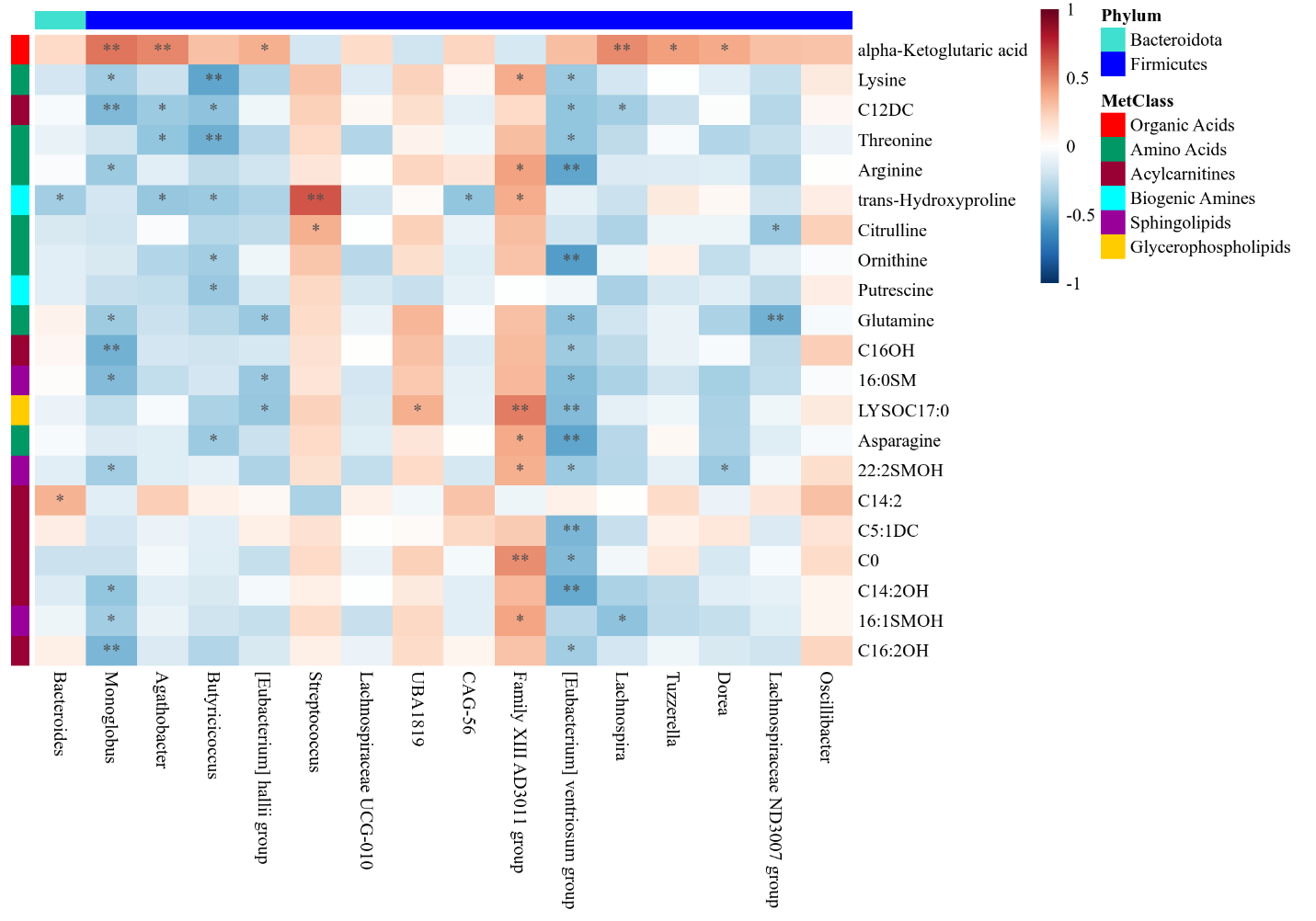


Supplementary Figure 31. Heatmap of Spearman correlations between differential microbes and metabolites at 9 months compared to baseline for sleeve gastrectomy.


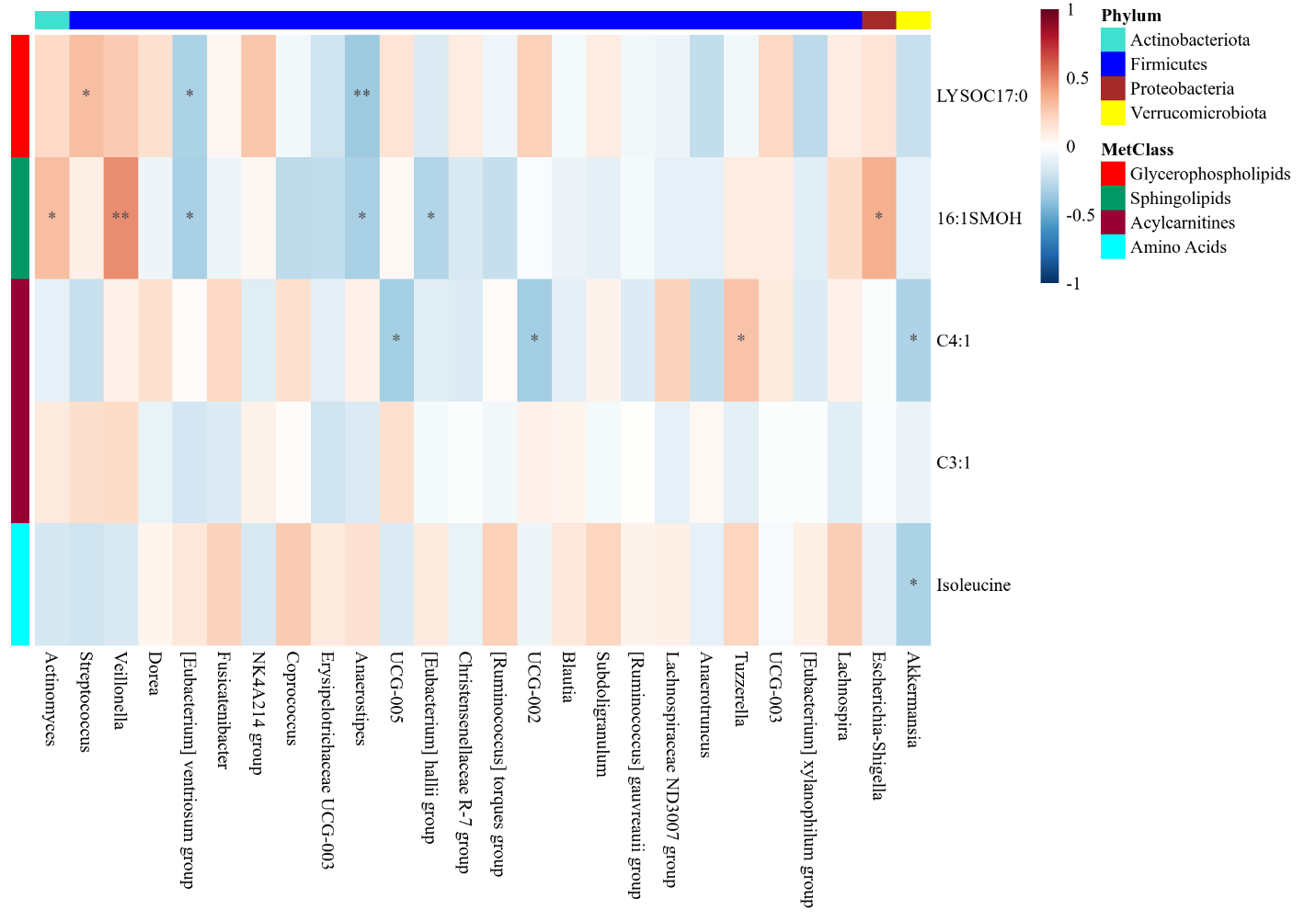


Supplementary Figure 32. Heatmap of Spearman correlations between differential microbes and metabolites at 3 months compared to baseline for Roux-en-Y gastric bypass.


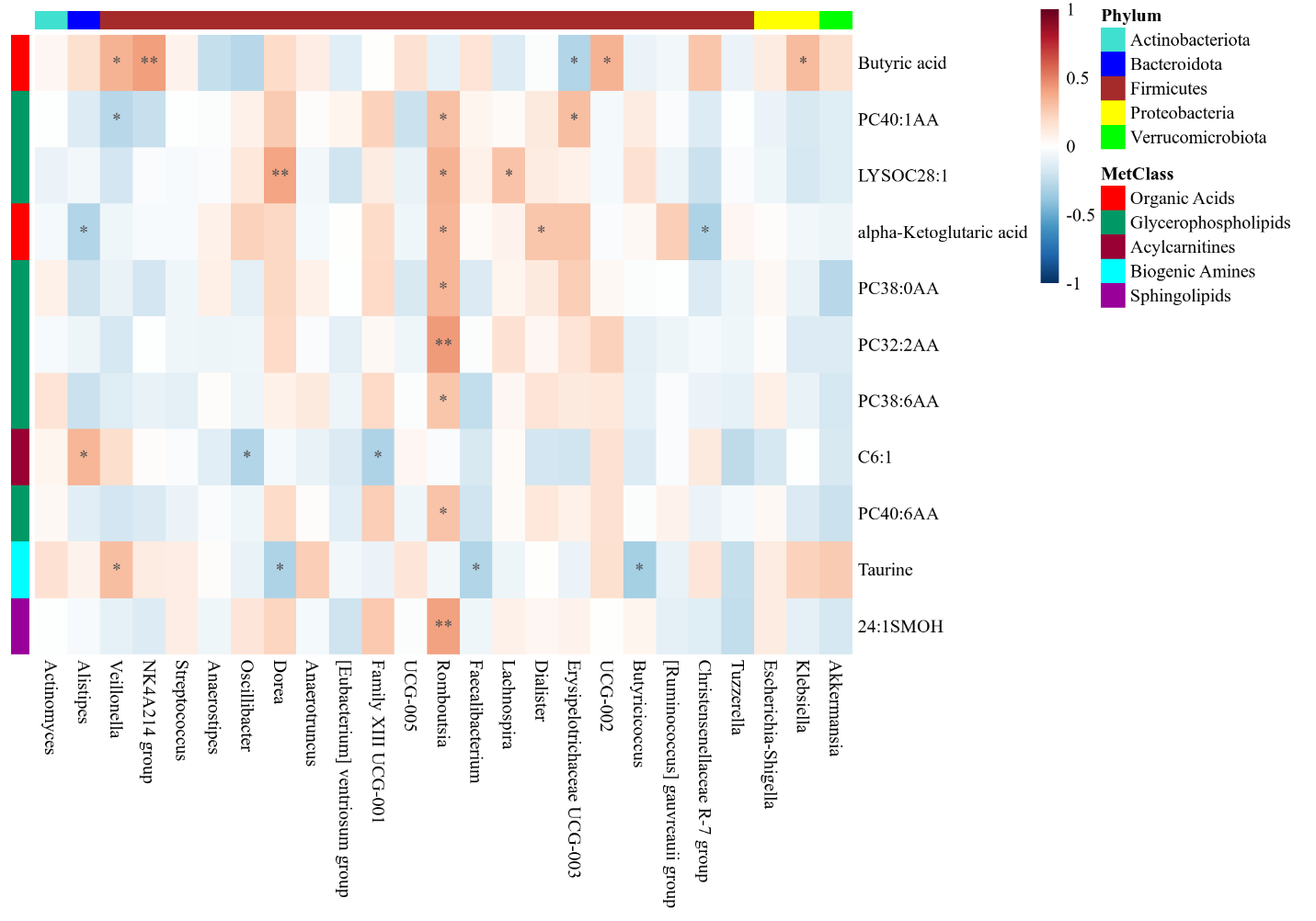


Supplementary Figure 33. Heatmap of Spearman correlations between differential microbes and metabolites at 9 months compared to baseline for Roux-en-Y gastric bypass.
